# Exosomes from M2c macrophages alleviate intervertebral disc degeneration by promoting synthesis of the extracellular matrix via MiR‐124/CILP/TGF‐β

**DOI:** 10.1002/btm2.10500

**Published:** 2023-02-16

**Authors:** Yi Liu, Mintao Xue, Yaguang Han, Yucai Li, Bing Xiao, Weiheng Wang, Jiangming Yu, Xiaojian Ye

**Affiliations:** ^1^ Department of Orthopaedics Second Affiliated Hospital of Naval Medical University Shanghai People's Republic of China; ^2^ Department of Orthopedics Tongren Hospital, Shanghai Jiao Tong University School of Medicine Shanghai People's Republic of China

**Keywords:** cartilage intermediate layer protein, exosomes, intervertebral disc degeneration, M2c polarization, macrophages, miRNAs

## Abstract

Immuno‐inflammation is highly associated with anabolic and catabolic dysregulation of the extracellular matrix (ECM) in the nucleus pulposus (NP), which dramatically propels intervertebral disc degeneration (IVDD). With the characteristics of tissue remodeling and regeneration, M2c macrophages have attracted great attention in research on immune modulation that rebuilds degenerated tissues. Therefore, we first demonstrated the facilitating effects of M2c macrophages on ECM anabolism of the NP in vitro. We subsequently found that exosomes from M2c macrophages (M2c‐Exoss) mediated their metabolic rebalancing effects on the ECM. To determine whether M2c‐Exoss served as positive agents protecting the ECM in IVDD, we constructed an M2c‐Exos‐loaded hyaluronic acid hydrogel (M2c‐Exos@HA hydrogel) and implanted it into the degenerated caudal disc of rats. The results of MRI and histological staining indicated that the M2c‐Exos@HA hydrogel alleviated IVDD in vivo in the long term. To elucidate the underlying molecular mechanism, we performed 4D label‐free proteomics to screen dysregulated proteins in NPs treated with M2c‐Exoss. Cartilage intermediate layer protein (CILP) was the key protein responsible for the rebalancing effects of M2c‐Exoss on ECM metabolism in the NP. With prediction and verification using luciferase assays and rescue experiments, miR‐124‐3p was identified as the upstream regulator in M2c‐Exoss that regulated CILP and consequently enhanced the activity of the TGF‐β/smad3 pathway. In conclusion, we demonstrated ameliorating effects of M2c‐Exoss on the imbalance of ECM metabolism in IVDD via the miR‐124/CILP/TGF‐β regulatory axis, which provides a promising theoretical basis for the application of M2c macrophages and their exosomes in the treatment of IVDD.

## 1 INTRODUCTION

Low back pain, numbness or paralysis of the lower limb, and spinal instability are severe clinical manifestations of spinal degenerative diseases, which is significantly prevalent with the aging of the population and places a heavy burden on social public health.^1^, ^2^, ^3^, ^4^ As the primary pathological condition of spinal degenerative diseases, intervertebral disc degeneration (IVDD) causes compression of the neural pathway, which leads to peripheral neural disorders.^5^, ^6^ The general treatment for IVDD‐caused spinal diseases is surgical resection of herniated discs to relieve neural compression, with solid interbody fusion and fixation. Although this surgical therapy generally achieves efficacious pain relief, it alters the distribution of stress on the spinal sequence and impairs intervertebral motion function, which increases the risk of adjacent segment disease.^7^, ^8^ Therefore, surgical discectomy and interbody fusion are not a perfect treatment for spinal degenerative disease.

The pathology of IVDD is highly related to the particular anatomical structure of the intervertebral disc. As the core of the intervertebral disc, the nucleus pulposus (NP) is a gelatinous tissue in which most water is anchored by a network skeleton of proteoglycan and type II collagen (Col II). Therefore, the NP effectively withstands and buffers the pressure load from the vertebral body.^9^ Under normal conditions, an isolation barrier composed of the annulus fibrosus and endplate keeps the NP isolated from the immune system.^10^ Minor damage to the cartilage endplate and annulus fibrosus caused by daily vertebral motion induces micro‐vascularization and the infiltration of inflammatory cells into the NP.^11^, ^12^ TNF‐α, IL‐1, and other pro‐inflammatory cytokines produced by inflammatory cells abnormally inhibit SOX‐9 expression, which further aggravates the apoptotic loss of NPCs, stimulates the oversecretion of metalloproteinases and results in the degeneration of the NP extracellular matrix (ECM).^13^, ^14^, ^15^, ^16^ Although NPCs can proliferate and remodel the ECM, cell vitality is impaired by disturbances in the metabolic and inflammatory microenvironments.^17^ To break through the current bottlenecks in IVDD therapy, it is critical to perform novel theoretical exploration on restoration of the self‐inflammation‐induced imbalance of matrix metabolism and activation of NP cells to the phenotype of ECM remodeling.

As vital immune‐regulating cells, macrophages may be polarized into distinct subtypes at different stages of inflammation.^18^, ^19^ Macrophages are polarized with LPS stimulation to the pro‐inflammatory M1 type in the early stage of inflammation, which serves as the initiator of inflammation to remove adverse substances that are hindering regeneration.^20^, ^21^, ^22^ Macrophages are stimulated by IL‐4 and polarized to the M2a type in the advanced stage of inflammation, which limits the extension of inflammation via the secretion of anti‐inflammatory cytokines to regulate inflammation. Macrophages stimulated by IL‐10 may be polarized into the M2c type, which contribute to tissue remodeling and regeneration.^23^, ^24^ The different types of macrophage polarization conversion are mutually transformed under distinct microenvironments.^23^ Dynamic maintenance of the temporal–spatial balance between different macrophage subtypes is beneficial to tissue regeneration.^18^ The infiltration of M1 macrophages positively correlated with the degree of IVDD, but the infiltration of M2‐type macrophages did not show a significant association with IVDD.^25^, ^26^ Therefore, the variant distribution of M1 and M2 macrophages in the intervertebral disc may be a special indicator of the progression of IVDD. M1 macrophages aggravate degeneration of intervertebral disc via activating HMGB1/Myd88/NF‐κB pathway and forming NLRP3 inflammasome,^27^ while M2a macrophages secrete CHI3L1 to promote ECM metabolic imbalance via ERK/JNK pathways, and transformed NPCs to a degenerative phenotype.^28^ Notably, no study investigated the effects of M2c macrophages on the metabolism and phenotype of NPCs.

Exosomes are nanoscale vesicles that are secreted autonomously by host cells into the intercellular space and absorbed by neighboring cells or distant tissue.^29^, ^30^ Exosomes transport the contained bioactive molecules to establish an informatic exchange between the original cells and recipient cells, which serve as a bridge for intercellular regulation.^31^ After fusing with recipient cells, exosomes release various cytokines, long noncoding RNAs and miRNAs derived from original cells, which affect the signaling pathway activation and transcription of recipient cells.^32^ Several studies found that exosomes played variable regulatory roles in promoting regeneration and delaying the aging of intervertebral discs. For example, exosomes derived from bone marrow mesenchymal stem cells effectively regulate endoplasmic reticulum stress in NP cells and inhibit their apoptosis.^33^, ^34^, ^35^ Although exosomes derived from M2 macrophages exert diverse effects in tissue degeneration, regeneration, and tumorigenesis,^36^ whether M2 macrophages affect NP metabolism and alter the process of IVDD via the exosomal transfer of key cytokines or miRNAs is not clear.

Based on the above review, we presumed that there was a potential relationship between the ECM metabolism of the NP and exosome‐mediated remodeling of M2c macrophages. To support this hypothesis, we first observed the influence of M2c macrophages and secreted exosomes on the vitality and metabolism of NPCs. We evaluated how the isolated exosomes from M2c macrophages improved matrix metabolism and the activity of NP cells to promote the regeneration of IVDD combined with hyaluronic acid (HA) hydrogel. We used proteomics to screen and verify the key miRNA/protein/pathway axis mediating the effects of M2c macrophage exosomes on the ECM‐related phenotype of the NP. The miR‐124‐enriched exosomes derived from M2c macrophages improved the metabolism of the NP matrix by inhibiting CILP in the present study, which activated the TGF‐β/smad3 signaling cascade and alleviated IVDD. This finding provides a theoretical and practical basis for IVDD therapeutic strategies based on the metabolic regulation of exosomes from immunomodulatory cells.

## 2 EXPERIMENTAL MATERIALS AND METHODS

The Ethics Committee of Naval Medical University approved all animal experiments, which were performed in accordance with the guidelines of the Institutional Animal Care and Use Ethics Committee of Naval Medical University.

### 2.1 Generation of M2c macrophages

Bone marrow‐derived macrophages (BMDMs) were prepared as described previously.^37^, ^38^ Briefly, BMDMs were extracted from the tibia and femur of 8‐week‐old Sprague–Dawley rats and cultured in RPMI‐1640 (Gibco) medium supplemented with 10% fetal bovine serum (Gibco), 1% penicillin–streptomycin (Gibco), and 10 ng/mL rat M‐CSF (Peprotech) in vitro. After incubation for 7 days, BMDMs without polarization (M0 macrophages) were obtained. M2c polarization of BMDMs was induced via the addition of 10 ng/mL recombinant Rat IL‐10 (Peprotech) o the medium and continuous incubation for 2 d.

### 2.2 Flow cytometry

To identify the M2c polarization of BMDMs, flow cytometry was performed to detect CD11b (a general marker of macrophages) and CD163 (a specific marker of M2c macrophages). After dissociation and resuspension, M2c macrophages were labeled with anti‐CD11b‐FITC and anti‐CD163‐PE (eBioscience) for 1 h. The positive rates of CD11b and CD163 on the surface of M2c macrophages were analyzed using flow cytometry (BD, USA).

### 2.3 Isolation and culture of rat NPCs

NP tissue was dissected from the coccygeal intervertebral disc of rats and cut into pieces. The tissues were digested in 0.2% type II collagenase (Gibco) for 3 h, filtered and washed in PBS. The isolated NPCs were centrifuged and resuspended in DMEM/F12 medium with 10% fetal bovine serum (Gibco) and 1% penicillin–streptomycin (Gibco). The culture medium was replaced every 3 days. NPCs at the third passage were used in the following procedures. In addition, for simulating pathological conditions of IVDD or stimulating TGF/smad3 pathway, recombinant Rat IL‐1β (Peprotech) and TNF‐α (Peprotech), or recombinant Human TGF‐β (Peprotech) were used to treated NPCs.

### 2.4 Isolation, identification, and internalization of exosomes from M2c macrophages

The M2c macrophages were incubated in medium with 5% exosome‐free FBS (SBI) for 48 h prior to exosome isolation. The medium of M2c macrophages was harvested and centrifuged at 2000 × *g* for 30 min at 4°C. After removing the residual debris, the supernatant was filtered through 0.22‐μm membrane filters and ultracentrifuged at 120,000 × g for 2 h using an Optima XPN‐90 ultracentrifuge (Beckman). The sediment was resuspended in 200 μL of precooled PBS and stored at −80°C.

The nanoparticle tracking analysis (NTA) for detecting the size of exosomes was performed using a ZetaView PMX 110 System (Particle Metrix). The morphology of exosomes was observed using a transmission electron microscope (JEM‐1400Flash). Specific markers of exosomes, including TSG101, CD9, and CD63, were tested using Western blotting.

To observe the internalization of exosomes by nucleus pulposus cells (NPCs), we added 2 μM PKH26 to the M2c‐Exos suspension and incubated it for 5 min at room temperature. The labeled exosomes were filtered through a 0.22‐μm filter and added to the medium of NPCs (3 × 10^4^ cells/well in a 24‐well plate). After incubating for 12 and 24 h, the NPCs were fixed with 4% polyformaldehyde, and their nuclei were stained with 4',6‐diamidino‐2‐phenylindole. The fluorescent exosomes in the cytoplasm of NPCs were photographed to show absorption into NPCs.

### 2.5 EdU staining

To assess the influence of M2c macrophages and their exosomes on the proliferation of NPCs, we seeded NPCs in 24‐well plates at a density of 1 × 10.^4^ After co‐culture with M2c macrophages in Transwell chambers (pore size: 0.4 μm) or treatment with M2c‐Exoss for 24 h, NPCs were incubated in medium with a 20 μM EdU working solution (5‐ethynyl‐2′‐deoxyuridine, Servicebio) for 6 h. After fixation with 4% polyformaldehyde, NPCs were incubated with an EdU fluorescent solution for 30 min in the dark. The percentage of EdU+ NPCs was calculated based on images obtained using fluorescence microscopy.

### 2.6 Cellular immunofluorescent staining

Prior to co‐culture with M2c macrophages or treatment with M2c‐Exoss, NPCs were cultured on slides at a proper density. NPCs were fixed, blocked with 4% polyformaldehyde and 5% BSA and incubated with primary anti‐Col II (Abcam, catalog: ab34712) and anti‐MMP13 (Abcam, catalog: ab39012) at 4°C overnight. NPCs were washed with PBS and incubated with the secondary fluorescent antibody in the dark for 2 h. After the nuclei of NPCs were stained with 4',6‐diamidino‐2‐phenylindole, we obtained fluorescent images using fluorescence microscopes (IX71; Olympus).

### 2.7 Migration of NPCs detected by crystal violet staining

To evaluate the influence of M2c macrophages and their exosomes on the migration of NPCs, we seeded NPCs on the polycarbonate membrane of Transwell chambers (pore size: 0.4 μm) at a density of 1 × 10.^4^ NPCs were co‐cultured with M2c macrophages or treated with M2c‐Exoss. Cell migration was detected at 12 and 24 h. After treatment, NPCs in the upper chamber were fixed with 4% polyformaldehyde and stained with 0.1% crystal violet (Servicebio) for 20 min. We used a cotton swab to wipe off the nonmigrated NPCs in the upper chamber. We used an optical microscope (Olympus, Japan) to obtain images for cell counting.

### 2.8 Western blotting

Proteins from NPCs and M2c‐Exoss were obtained using RIPA lysis buffer (Servicebio, China) and quantified using a BCA assay. Proteins were separated using SDS‐PAGE and transferred to a polyvinylidene fluoride membrane (Servicebio, Wuhan, Hubei, China). Membranes were blocked with TBST buffer containing 5% skim milk for 1 h and incubated with the following primary antibodies overnight at 4°C: anti‐TSG101 (Abcam, Cambridge, UK, catalog: ab125011), anti‐CD9 (Abcam, catalog: ab236630), anti‐CD63 (Abcam, catalog: ab134045), anti‐Col II (Abcam, catalog: ab34712), anti‐aggrecan (Abcam, catalog: ab36861), anti‐MMP13 (Abcam, catalog: ab39012), anti‐ADAMTS5 (Abcam, catalog: ab41037), anti‐β‐actin (Cell Signaling Technology, catalog: #4970), anti‐CILP (Thermo Fisher, catalog: PA5‐18553), anti‐Smad3 (Cell Signaling Technology, catalog: #9513), and anti‐phosphorylated Smad3 (Cell Signaling Technology, catalog: #9520). The membranes were incubated with HRP‐conjugated secondary antibodies for 1.5 h. Protein bands were visualized using an ECL kit (Servicebio), and gray values were quantified using ImageJ software.

### 2.9 Fabrication and release detection of the M2c‐Exos@HA hydrogel

First, 1 g of HA powder (Sigma, catalog: 924474) was dissolved with then 100 mL of deionized water and evenly stirred on a magnetic stirrer. Then 1 mM hydrochloric acid solution was slowly added to the prepared HA solution until its pH dropped to about 4.7. Afterward, 0.4 g of adipate diphthalide was added to the solution and mixed evenly, then 0.4 g EDCI was added and stirred for 30 minutes. After its pH was adjusted to about 7 by slowly adding 1 mM sodium bicarbonate solution, HA solution became hydrogel. After being on dialysis for 2 days, HA hydrogel was preserved by rapid freezing and drying. To fabricate HA hydrogels containing M2c‐Exoss, 0.01 g of freeze‐dried HA hydrogel and 1 mL suspension of M2c‐Exoss (0.5 mg/mL) mixed and kept stirring in an ice bath for 30 min.

The internal morphology and structure of HA hydrogel were observed using scanning electron microscopy (S‐3400N, **Hitachi**). The storage modulus (*G*′), loss modulus (*G*′′) and viscosity of M2c‐Exos@HA hydrogel was measured by rheological test (Physica MCR302, Anton Paar).

Before the construction of the M2c‐Exos@HA hydrogel, PKH26 was used to label M2c‐Exoss as mentioned above. The fluorescence intensity on the surface of the M2c‐Exos@HA hydrogel was observed under a fluorescence microscope at different time points (0, 1, 3, 6, 12, and 24 h) to reveal the release of M2c‐Exoss from the HA hydrogel in the short term.

To test the long‐term cumulative release of M2c‐Exoss from the HA hydrogel, 0.5 mL per well of the M2c‐Exos@HA hydrogel was placed in a 24‐well plate and covered with 0.5 mL PBS buffer. The PBS buffer above the M2c‐Exos@HA hydrogel was collected and restored at different time points (2‐day intervals for 60 days). The cumulative release curve of M2c‐Exoss was tested using the BCA method.

### 2.10 Animal experiment

Eighty Sprague–Dawley rats (12 weeks old, male) were randomly divided into four groups: sham group (*n* = 20), IVDD group (*n* = 20), HA hydrogel group (*n* = 20), and M2c‐Exos@HA hydrogel group (*n* = 20). Rats were anesthetized via an intraperitoneal injection of 2% pentobarbital (0.3 mL/100 g weight), fixed on the operating platform, and their tails were sterilized with ethanol. The segment of the caudal intervertebral disc (Co9/10) was percutaneously punctured at a depth of 5 mm with a 20‐gauge needle and rotated for 30 s. For the sham group, the puncture depth was approximately 0.5 mm, which maintained annulus fibrosus integration. At 2 weeks post‐initial surgery when IVDD was established, rats in each group were anesthetized again and received corresponding injections in the degenerated segment of caudal intervertebral disc: 5 μL PBS for IVDD group; 5 μL HA hydrogel for HA hydrogel group; and 5 μL M2c‐Exos@HA hydrogel for M2c‐Exos@HA hydrogel group. This puncture injection was performed using a 10‐μL micro‐syringe.

### 2.11 In vivo tracing of M2c‐Exos

To trace M2c‐Exoss in the caudal intervertebral discs of rats, M2c‐Exoss were labeled with PKH26 as described earlier. The labeled M2c‐Exoss were suspended in PBS or mixed into HA hydrogel and observed using an in vivo imaging system (IVIS Lumina LT Series III) at different time points (0.5, 1, 3, 7, 14, and 28 days).

### 2.12 MRI examination

The present study used a 7.0‐T magnetic resonance imaging (MRI) system (Bruker BioSpec 7T/20 USR; Bruker AXS GmbH, Karlsruhe, Germany) to scan the caudal intervertebral disc of rats 4 and 8 weeks after exosomal intervention. After gas anesthesia, the rats were placed on the MRI platform, and the Co8/9, Co9/10, Co10/11 segments of the intervertebral discs were located. T2‐weighted phase sections in sagittal and cross‐sectional plane were acquired with the following settings: repetition time 2500 ms, echo time 16 ms; field of view 3.0 cm; and layer thickness 0.7 mm. The obtained MRI images were analyzed using Radiant Dicomviwer software. The T2 signal intensity in the NP was measured, and the MRI index of the NP was calculated to assess the degree of IVDD.

### 2.13 Histological staining

Four and 8 weeks after intervention, the rats were sacrificed, and the Co9/10 intervertebral discs were harvested. Discs were fixed, decalcified, and embedded into paraffin blocks. Sagittal sections of IVDD samples at a thickness of 5 μm were obtained using radial microtomes and fixed on slides. Hematoxylin & eosin and safranin‐O/fast green staining was performed to stain all slides according to the manufacturer's instructions. An optical microscope (Olympus, Japan) was used to obtain histological images. According to a previous study,^39^ a scoring system for grading the histological degeneration of intervertebral discs was applied in the present study. Briefly, the cellularity and morphology of intervertebral disc were evaluated by calculating scores from such aspects: count and morphology of cells in NP, count of fibroblast and morphology of fibers in annulus fibrosus, and the morphology of border between NP and annulus fibrosus. Higher histological scores significantly predicted worse IVDD.

### 2.14 Immunohistochemical staining

To evaluate the expression of Col II and MMP13 in the discs of each group of tissues, the sections were incubated with 0.01 M citrate buffer for 15 min at 95°C and blocked with 5% skimmed milk for 30 min at 37°C. Tissue sections were incubated at 4°C overnight with primary antibody (anti‐Col II, 1:200, Abcam; MMP13, 1:500, Abcam). The sections were incubated with HRP‐conjugated secondary antibodies (Abcam) for 1 h at 37°C. The nucleus was stained with hematoxylin. IHC images were obtained using an optical microscope (Olympus). The IHC Profiler of ImageJ software was used to determine the score of the positive staining area.^40^

### 2.15 4D label‐free proteomics

#### 2.15.1 Sample Preparation

NPCs were treated with M2c‐Exoss or M0‐Exos (150 μg/mL) for 48 h and lysed using SDT buffer. The lysate was sonicated and boiled for 15 min. After centrifugation at 14,000 × *g* for 40 min, the supernatant was quantified using the BCA method. Proteins from NPCs were prepared as peptides for subsequent mass analysis in FASP digestion.^41^

#### 2.15.2 Mass spectrometry analysis

Peptides were analyzed on a nanoElute (Bruker, Bremen, Germany) coupled to a timsTOF Pro (Bruker). Through a 25 cm × 75 μm analytical column and 1.6 μm C18 beads with a packed emitter tip (IonOpticks, Australia), peptides were separated at 300 nL/min using a linear gradient as follows: 3% buffer B for 3 min, 3%–28% buffer B for 70 min, 28%–38% buffer B for 7 min, 38%–100% buffer B for 5 min, and hold in 100% buffer B for 5 min. The peptides were analyzed using a timsTOF Pro system (Bruker) in parallel accumulation serial fragmentation (PASEF) mode: mass range 100–1700 m/z, 1/K0 start 0.6 V·s/cm^2^ end 1.6 V·s/cm^2^, ramp time 100 ms, lock duty cycle to 100%, capillary voltage 1500 V, dry gas 3 L/min, and dry temperature 180°C. The following PASEF settings were used: 10 MS/MS scans (total cycle time 1.16 s), charge range 0–5, active exclusion for 0.4 min, scheduling target intensity 20,000, intensity threshold 2500, and CID collision energy 42 eV.

#### 2.15.3 Data analysis

The MS data were analyzed using MaxQuant software (Version 1.6.14.0). All peptide sequences were aligned to the NCBInr database downloaded from NCBI (ncbi‐blast‐2.2.28 + −win32.exe), and only the sequences in the top 10 with an E‐value ≤ 1 e−3 were retained. The cutoff of the global false discovery rate (FDR) for peptide and protein identification was set to 0.01. Protein abundance was calculated on the basis of the normalized spectral protein intensity (LFQ intensity). Proteins with |Fold change| >2 and adj‐*p* value <0.05 were considered differentially expressed proteins. The GO term of proteins with top Bit‐Score in Blast2GO was selected. The annotation from GO terms to proteins was completed using Blast2GO Command Line. After elementary annotation, InterProScan was used to add the functional motif information to proteins to improve annotation. Pathway analysis was performed using the KEGG database. Fisher's exact test was used to enrich GO terms and pathways.

### 2.16 Real‐time quantitative PCR

Total miRNAs from NPCs and M2c‐Exoss were collected using an Exosome DNA/RNA Extraction Kit (Guidechem). We used cDNA synthesis kits (Toyobo, Osaka, Japan) to reverse transcribe cDNA. We performed real‐time PCR in a StepOnePlus real‐time PCR system (Applied Biosystems) using SYBR Green PCR Master mix (Applied Biosystems). The 2−ΔΔCT method was used to evaluate the relative expression of miRNAs. The primers used in the present study are listed in Table S1.

### 2.17 Transfection of plasmid

Plasmids containing the pcDNA 3.1‐CILP sequence, miR‐124 inhibitor or negative control vector were constructed by GenePharma (Shanghai). NPCs were seeded on 24‐well plates at a density of 4 × 10^4^ cells/well and incubated until they reached 50% confluence. We transfected NPCs with the plasmid using Lipofectamine® 2000 (Invitrogen) for 48 h according to the manufacturer's instructions. The effect of CILP overexpression was validated using Western blot analysis.

### 2.18 Luciferase reporter assay

To construct the CILP 3′‐UTR reporter, the 3′‐UTR sequence (WT), and miR‐124 binding mutant sequence (MUT) of CILP mRNA were synthesized and cloned into the pmirGLO luciferase vector (Promega, Madison, WI, USA) by GenePharma (Shanghai) (pmirGLO‐WT‐CILP and pmirGLO‐MUT‐CILP, respectively). HEK293 cells (ATCC) were transfected with miR‐NC OE, and miR OE and seeded in 12‐well plates at a density of 3 × 10^5^ cells/well. The cells were transfected with pmirGLO‐WT‐TLR4 or pmirGLO‐MUT‐TLR4 using Lipofectamine® 2000 (Invitrogen) for 48 h. The cells were treated with miR‐124 mimics for 48 h via transfection with plasmid. Luciferase activity assessment was performed using a dual luciferase reporter detection kit (Promega).

### 2.19 Statistical analysis

The measurement data are presented as the mean ± standard deviation. SPSS (Version 25.0, IBM, USA) was used to perform statistical analyses. For data with a normal distribution, Student's *t* test, one‐way, or two‐way ANOVA was used for comparisons. For data without a normal distribution, the Mann–Whitney U test was used for comparisons. A *p* value <0.05 was selected as the cut‐off for statistical significance.

## 3 RESULTS

### 3.1 M2c macrophages enhanced the proliferation, migration, and ECM synthesis of NPCs

**FIGURE 1:**
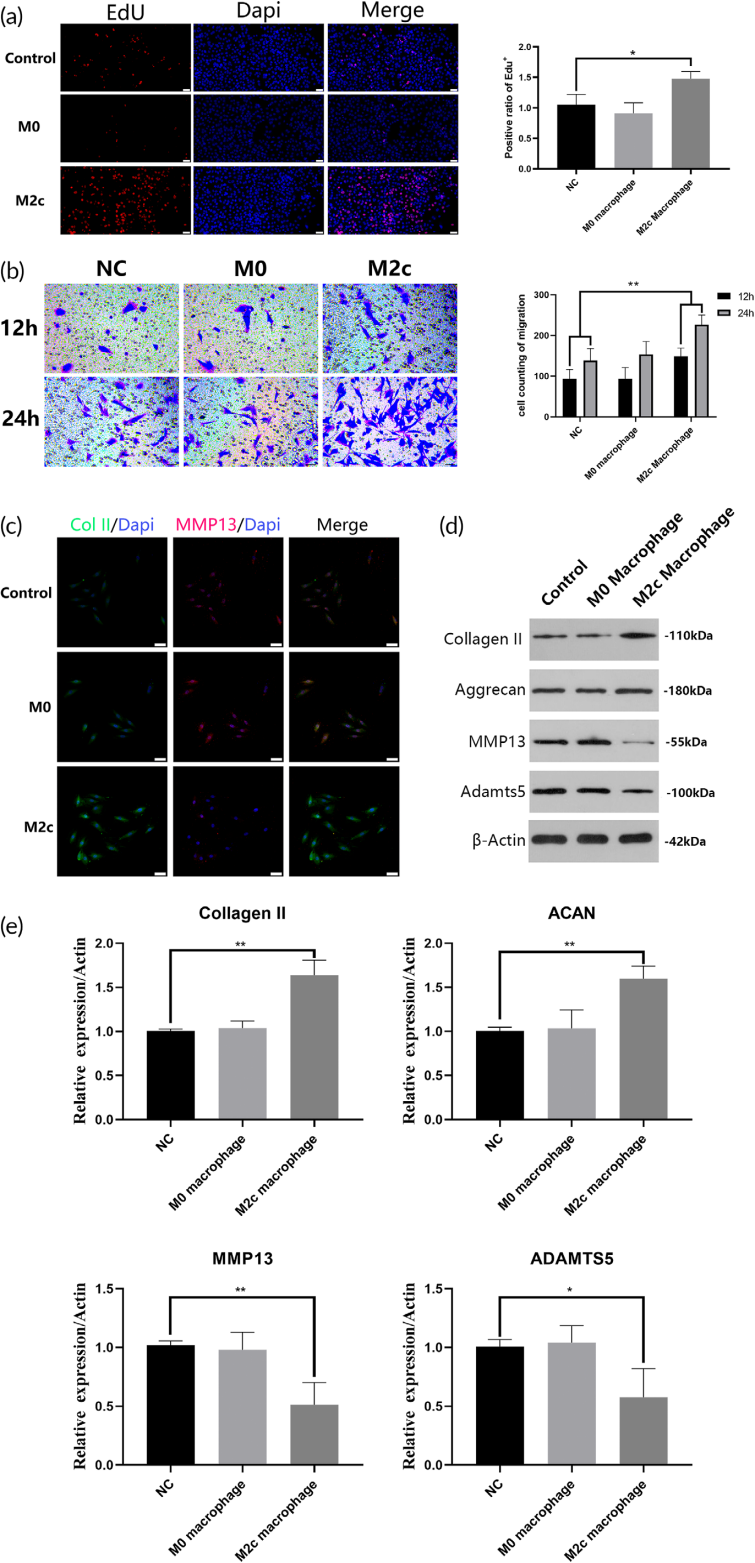
Paracrine effects of M2c macrophages on nucleus pulposus cells (NPCs) in vitro. (a) The proliferation of NPCs co‐cultured with M2c macrophages was detected using EdU staining. (b) Migration of NPCs co‐cultured with M2c macrophages was detected using crystal violet staining at 12 and 24 h. (c, d) The expression of Col II, aggrecan, MMP13, and ADAMTS5 in NPCs co‐cultured with M2c macrophages was assessed using immunofluorescent staining and Western blotting. Scale bar = 100 μm. *p < 0.05, **p < 0.01.

First, M0 macrophages were polarized into M2c macrophages by IL‐10 and TGF‐β. Immunofluorescence staining revealed that the expression of CD11b (pan‐marker for macrophage) was similar between M2c and M0 macrophage, while the expression of CD163 (specific marker for M2c macrophage) was significantly elevated in M2c macrophages, compared with that in M0 macrophages (Figure S1A). Flow cytometry showed consistent difference in expression of CD163 between M2c and M0 macrophages (Figure S1B). After successful polarization had been identified, we established a co‐culturing system of M2c macrophages in the top chamber and NP cells in the bottom chamber for 24 h. EdU staining was used to investigate the influence of M2c macrophages on the vitality of NPCs in vitro. As shown in Figure 1a, the EdU fluorescence intensity of NPCs was significantly increased under M2c macrophage co‐culturing compared to the control group. We re‐cultured NPCs with M2c macrophages in the Transwell system (NPCs in the upper chamber and M2c macrophages in the bottom chamber) to observe the effect of M2c macrophages on the migration of NPCs. Figure 1b shows that the number of NPCs positively stained with crystal violet in the M2c macrophage co‐culturing group was significantly higher than the M0 macrophage co‐culturing group after 12 and 24 h. These results indicated that M2c macrophages significantly enhanced the proliferation and migration of NPCs.

To examine whether M2c macrophages affected the expression of ECM proteins (Col II and aggrecan) and metalloproteinases (MMP13 and ADAMTS5) in NPCs, we extracted proteins from NPCs co‐cultured with M2c macrophages for 48 h and detected the proteins using immunofluorescence staining and Western blotting. Figure 1c–e shows that the expression of Col II and aggrecan in NPCs co‐cultured with M2c macrophages was significantly up‐regulated compared to the control group, and the expression of MMP13 and ADAMTS5 was significantly down‐regulated. This finding indicated that M2c macrophages regulated the balance of ECM metabolism and promoted the secretion of matrix proteins in NPCs.

### 3.2 Inhibition of exosome secretion impeded the effects of M2c macrophages on ECM synthesis in NPCs

**FIGURE 2:**
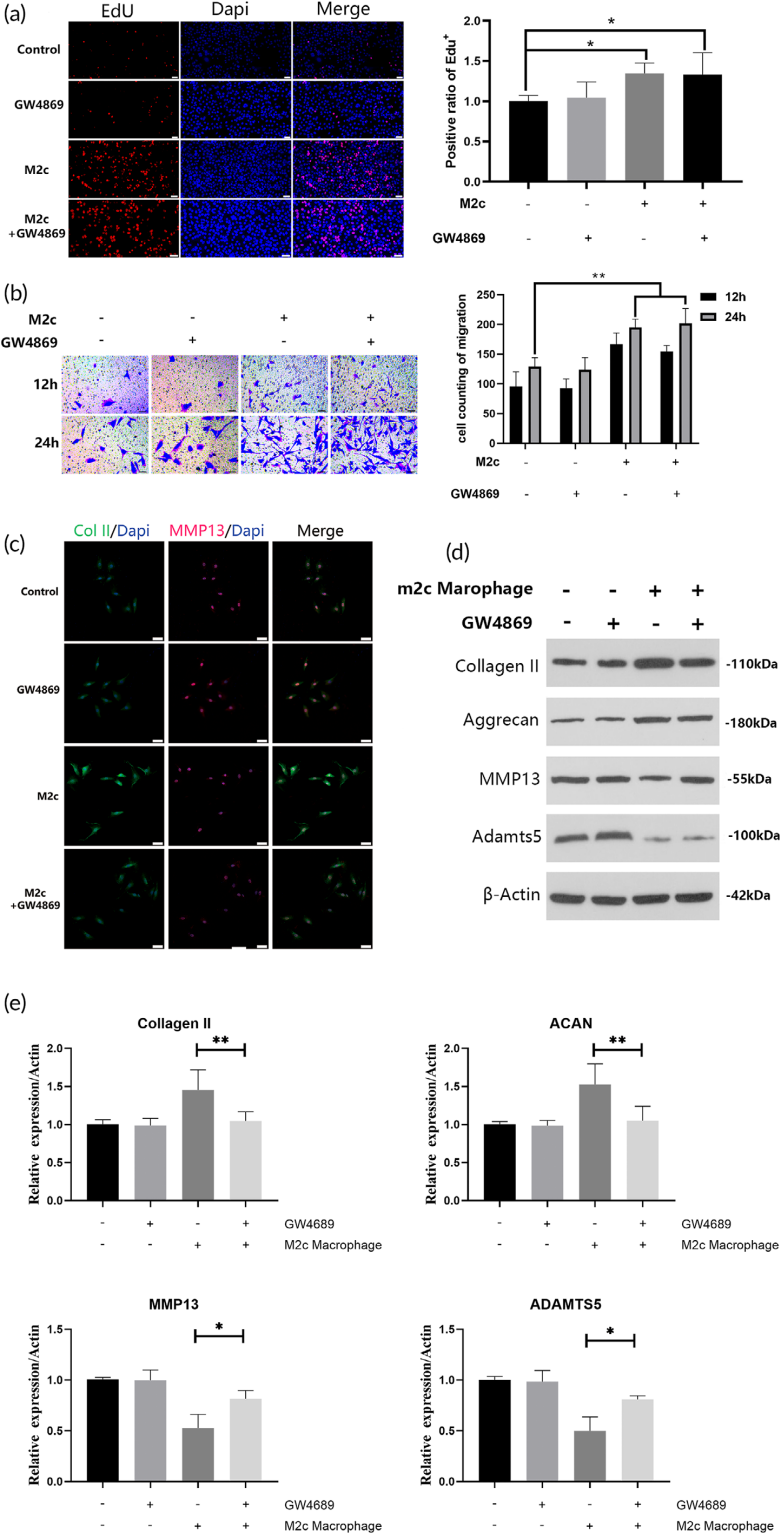
Pretreatment with GW4869 impaired the promoting effects of M2c macrophages on extracellular matrix (ECM) synthesis of nucleus pulposus cells (NPCs). (a) Proliferation of NPCs co‐cultured with M2c macrophages pretreated with GW4869 was detected using EdU staining. (b) Migration of NPCs co‐cultured with M2c macrophages pretreated with GW4869 was detected using crystal violet staining at 12 and 24 h. (c, d) The expression of Col II, aggrecan, MMP13, and ADAMTS5 in NPCs co‐cultured with M2c macrophages pretreated with GW4869 was assessed using immunofluorescent staining and Western blotting. Scale bar = 100 μm. *p < 0.05, **p < 0.01.

Various cytokines and exosomes relay intracellular effects via a paracrine mechanism. Therefore, we indirectly examined whether M2c macrophages stimulated the self‐renewal, migration, and ECM metabolic regulation of NPCs by delivering exosomes. The present study pretreated M2c macrophages with the exosome‐secreting inhibitor GW4869 for 24 h then co‐cultured the cells with NPCs to observe changes in proliferation and migration. Figure 2a,b shows that regardless of GW4869 pretreatment, M2c macrophages still significantly increased the EdU fluorescence intensity and crystal violet positive rates of NPCs. There was no significant difference in the proliferation or migration of NPCs between the pretreated and nonpretreated group groups. Therefore, these results indicated that the mechanism by which M2c macrophages promote the proliferation and migration of NPCs was independent of their derived exosomes, which may be mediated by other paracrine cytokines.

We further examined the effect of GW4869‐pretreated M2c macrophages on the ECM metabolism of NPCs. As shown in Figure 2c–e, although the expression of Col II and aggrecan in NPCs was higher in the co‐culturing group in which M2c macrophages were pretreated with GW4869 than the control group (no macrophage co‐culture), it was significantly lower than in the co‐culturing group in which M2c macrophages were not pretreated. The expression of MMP13 and ADAMTS5 in NPCs co‐cultured with pretreated M2c macrophages exhibited a similar dysregulated pattern, which was significantly higher than the control group and lower than the nonpretreated group. Therefore, after inhibition of exosomal secretion, M2c macrophages had weaker stimulating effects on the expression of matrix proteins and inhibiting effects on the expression of metalloproteinases in NPCs. These results indirectly indicate that M2c macrophages may promote the secretion of ECM proteins and inhibit metalloproteinases in NPCs by secreting exosomes.

### 3.3 Extraction and identification of exosomes from M2c macrophages

The present study isolated exosomes from the median supernatant of M2c macrophages (M2c‐Exoss) using ultracentrifugation and characterized the exosomes using transmission electron microscopy (TEM), NTA, and Western blotting. First, the expression of the exosome markers CD9, CD63, and TSG101 was verified using Western blotting (Figure S2A). TEM revealed that the exosomes were morphologically intact and uniform in size and showed typical round shapes (Figure S2B). Finally, NTA (Figure S2C) showed that the diameter of M2c‐Exoss appeared to be normally distributed and reached a single peak at 125 nm, which was consistent with the feature size of exosomes. These results suggested that the M2c‐Exoss isolated by ultracentrifugation conformed to the normal morphological characteristics of general exosomes.

We labeled M2c‐Exoss with PKH26 to test internalization by NPCs. After incubation with M2c‐Exoss for 24 and 48 h, the red fluorescence of PKH26 was significantly enriched in the cytoplasm of NPCs, which indicated that M2c‐Exoss were absorbed into NPCs (Figure S2D).

### 3.4 M2c‐Exoss mimicked the function of M2c macrophages on ECM synthesis of NPCs in a concentration‐dependent manner

**FIGURE 3:**
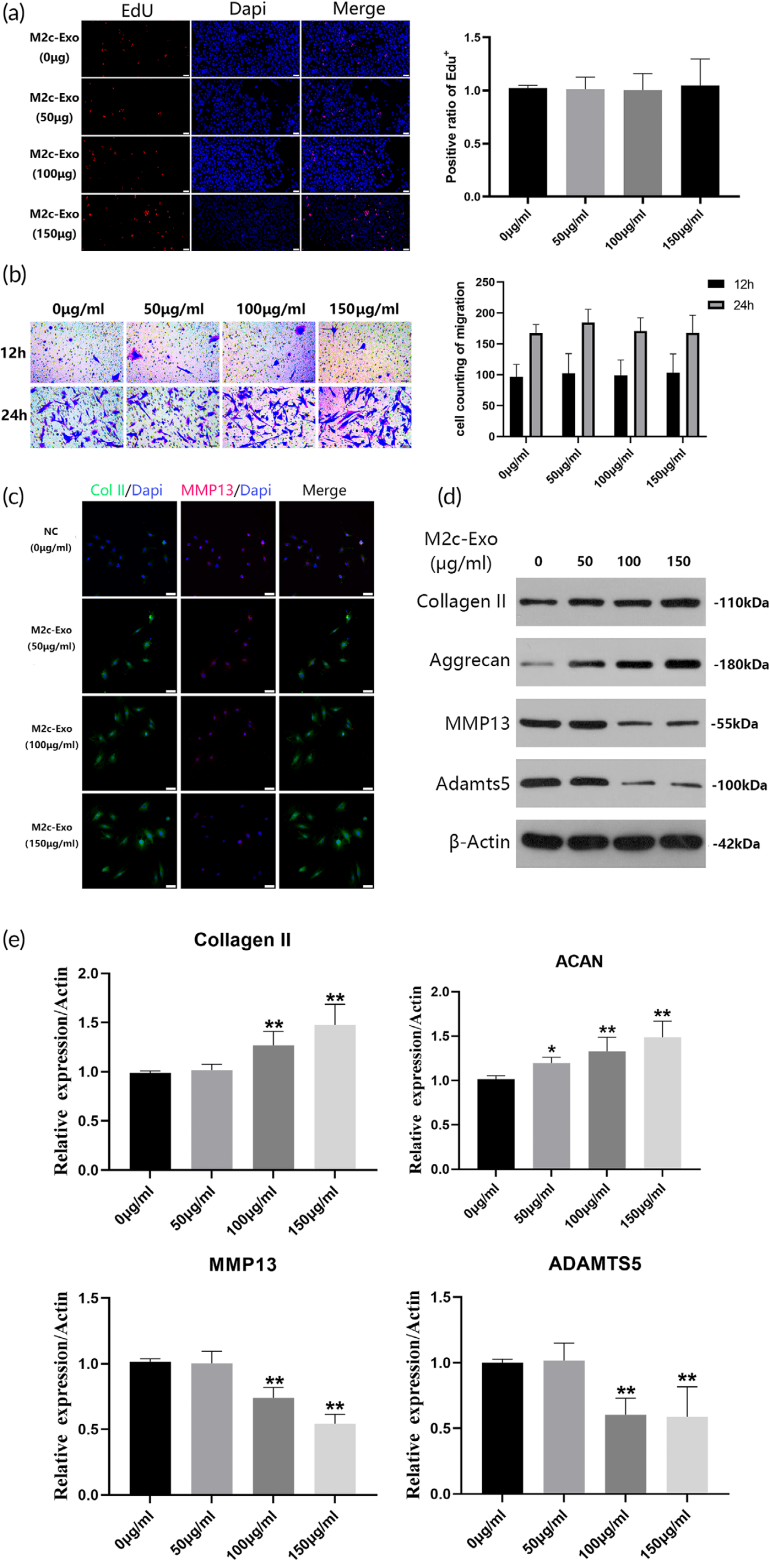
M2c‐Exoss enhanced extracellular matrix (ECM) synthesis of nucleus pulposus cells (NPCs). (a) The proliferation of NPCs treated with M2c‐Exoss (0, 50, 100, and 150 μg/mL) was detected using EdU staining. (b) Migration of NPCs treated with M2c‐Exoss (0, 50, 100, and 150 μg/mL) was detected using crystal violet staining at 12 and 24 h. (c, d) The expression of Col II, aggrecan, MMP13, and ADAMTS5 in NPCs treated with M2c‐Exoss (0, 50, 100, and 150 μg/mL) was assessed using immunofluorescent staining and Western blotting. Scale bar = 100 μm. *p < 0.05, **p < 0.01.

To directly demonstrate the phenotypic effects of M2c‐Exoss on the viability of NPCs, the cells were treated with M2c‐Exoss at different concentrations (0, 50, 100, and 150 μg/mL). After incubation for 24 h, EdU staining showed that the number of proliferating NPCs was not significantly different between the M2c‐Exos‐treated group and the control group (Figure 3a). Figure 3b shows that after 12 and 24 h of continuous intervention, different concentrations of M2c‐Exoss did not significantly influence the positive rate of crystal violet staining in NPCs. These results indicated that M2c‐Exoss did not stimulate the proliferation or migration of NPCs, which was consistent with the results of GW4869 pretreatment mentioned above.

We treated NPCs with M2c‐Exoss at the same concentration to investigate how M2c‐Exoss regulated the expression of ECM proteins and metalloproteinases. As shown in Figure 3c–e, the expression of Col II and aggrecan in NP cells increased significantly with increasing concentrations of M2c‐Exoss. The expression of MMP13 and ADAMTS5 in NPCs showed a significant and opposite trend to the ECM proteins and decreased with increasing M2c‐Exos concentrations. The effects of M2c‐Exoss on ECM synthesis in NPCs reached a maximum at 150 μg/mL. These results indicated that M2‐Exos promoted the expression of ECM proteins and inhibited the synthesis of metalloproteinases in NPCs in a concentration‐dependent manner.

Moreover, we intended to investigate the potential effects of M2c‐Exoss on ECM metabolism of NPCs in pathological conditions of IVDD. For simulating the pathological microenvironment in vitro, pro‐inflammatory cytokines, IL‐1β (10 ng/mL), and TNF‐α (25 ng/mL), were used to treat NPCs for 48 h, combined with or without 150 μg/mL of M2c‐Exos. As shown in Figure S3, M2c‐Exoss can significantly mitigate the downregulating effects of TNF‐α and IL‐1β on Col II and Aggrecan expression and upregulation on MMP13 and Adamts5 in NPCs. This means the M2c‐Exoss also exert protective effect on ECM metabolism of NPCs in pathological conditions of IVDD.

### 3.5 Characterization of HA hydrogel loaded with M2c‐Exoss

**FIGURE 4:**
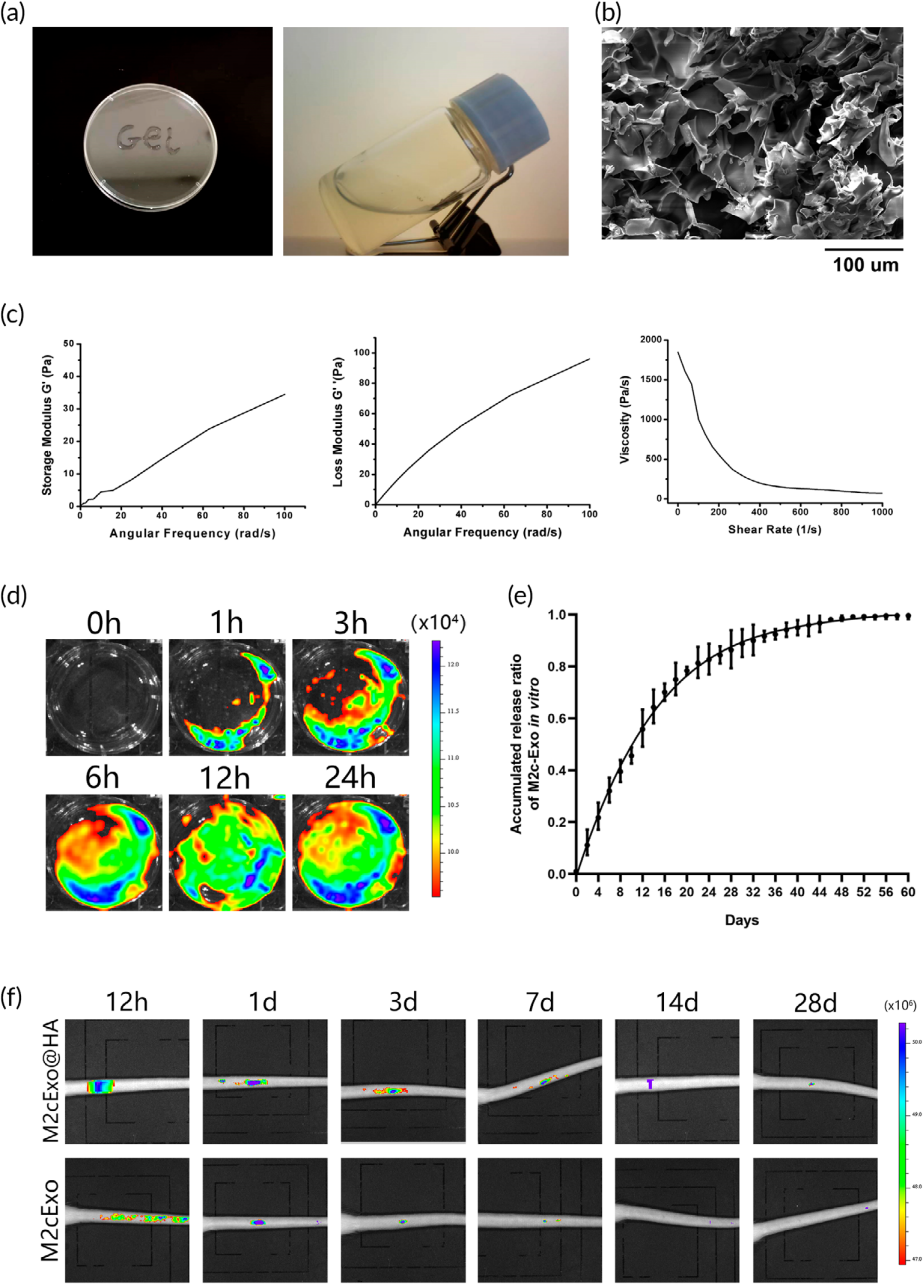
Characteristics of HA hydrogels loaded with M2c‐Exoss. (a) A photograph of the M2c‐Exos@HA hydrogel. (b) SEM image of M2c‐Exos@HA hydrogel. (c) Rheological analysis of M2c‐Exos@HA hydrogel. (d) The release of PKH‐26‐labeled M2c‐Exoss from HA hydrogels in 24 h. (e) Cumulative curve of M2c‐proteins released from HA hydrogels assessed using the BCA assay. (f) Retention of M2c‐Exoss loaded with HA hydrogels in caudal discs of rats at different time points after implantation.

Hyaluronic acid (HA) hydrogels have excellent biocompatibility and capacity of loading and transferring biomolecules in vivo application. Given the rapid clearance of exosomes in vivo, we chose HA hydrogels as carriers for encapsulating exosomes. The general view of M2c‐Exos@HA hydrogel was shown in Figure 4a. The internal morphology of M2c‐Exos@HA hydrogel was characterized with three‐dimensional porous network structure, in which M2c‐Exoss adhered to the inner surface of the HA hydrogel (Figure 4b). The elastic modulus of M2c‐Exos@HA hydrogels was studied by oscillatory rheological experiment. As shown in Figure 4c, the storage modulus (*G*′) and loss modulus (*G*′′) of M2c‐Exos@HA hydrogels increased uniformly with the increase of angular frequency, which indicated it was stable viscoelastic gel. In addition, the viscosity of M2c‐Exos@HA hydrogels decreased with the increase of shear rate (Figure 4c), which was consistent with the storage modulus (*G*′) and loss modulus (*G*′′) of hydrogels, confirming that hydrogel had eligible rheological properties.

To evaluate whether the HA hydrogel had the advantage of a slow M2c‐Exos release, we constructed HA hydrogels loaded with PKH26‐labeled M2c‐Exoss, and the short‐term release of M2c‐Exoss from HA hydrogels was observed in vitro. The fluorescence on the surface of the HA hydrogel gradually increased within 24 h of loading with M2c‐Exoss (Figure 4d). We continuously detected for 60 days and plotted the cumulative curve of M2c‐Exos protein to reflect their cumulative release from HA hydrogel. As shown in Figure 4e, the efficiency of M2c‐Exos release was maximal 7 days after gelation of the hydrogel (the slope of the tangent line in the cumulative curve at 7 days was the highest) and gradually slowed at 16 days. The ratio of aggregate M2c‐Exos release reached 90% at 35 days after gelation. This result suggested that the HA hydrogel can continuously release M2c‐Exoss for approximately 6 weeks in vitro.

To probe the retention of M2c‐Exoss loaded in HA hydrogel in vivo, we punctured the coccygeal discs of rats to establish an animal model of IVDD and injected PKH26‐labeled M2c‐Exos@HA hydrogels into the punctured disc in a volume of 5 μL. The same volume of PKH26‐labeled M2c‐Exoss was injected into the control group. In vivo imaging showed (Figure 4f) that the fluorescence signal of M2c‐Exoss without HA hydrogel decreased significantly 3 days after injection and completely disappeared at 14 days, but the fluorescence signal of M2c‐Exoss loaded in HA hydrogel was maintained until 28 days after injection. This finding indicated that the HA hydrogel delayed the metabolic clearance rate of M2c‐Exoss in vivo and significantly prolonged the effective time of M2c‐Exoss in intervertebral discs, which is helpful to provide essential stimulation for the regeneration of IVDDs.

### 3.6 M2c‐Exoss@HA hydrogel alleviated IVDD by promoting ECM synthesis and inhibiting its degradation in vivo

**FIGURE 5:**
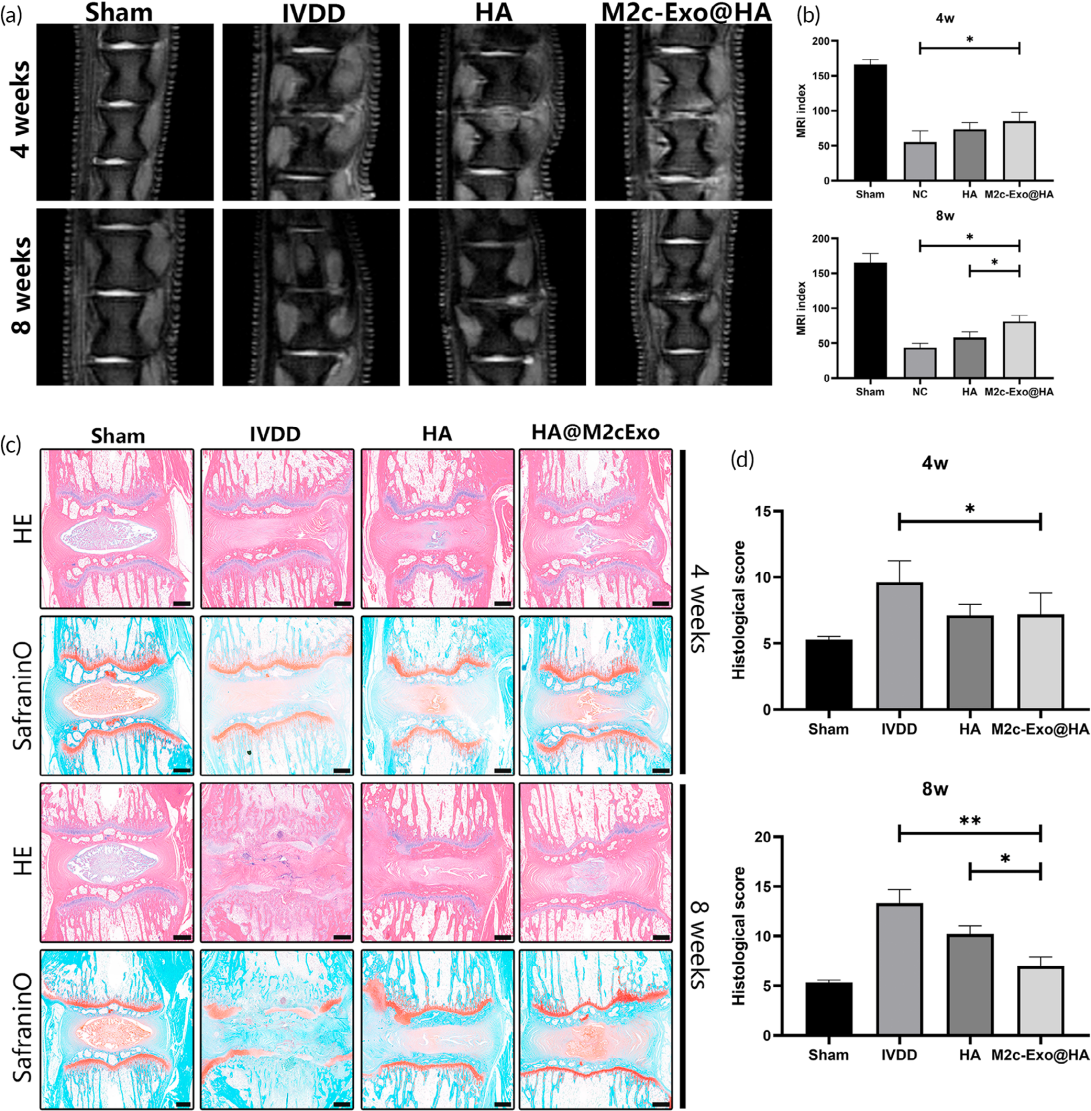
Administration of the M2c‐Exos@HA hydrogel alleviated IVDD in vivo. (a) Representative MRI scans of caudal vertebral discs 4 and 8 weeks after implantation in different groups. (b) Quantification of MRI scans in various groups using the MRI index. (c) Longitudinal H&E and safranin O staining of caudal vertebral discs in different groups 4 and 8 weeks after implantation. (d) Comparison of IVDD histological scores between different groups. *p < 0.05, **p < 0.01. Scale bar: 200 μm.

To assess the therapeutic effects of M2c‐Exoss combined with HA hydrogels on IVDD, we injected PBS, HA, and M2c‐Exos@HA into the degenerated coccygeal disc 4 weeks after establishment of the IVDD model. We performed MRI examination 4 and 8 weeks after injection and measured the MRI index, which reflected the change in T2 signals and structure during the development of IVDD. As shown in Figure 5a,b, the MRI index of the degeneration group was 55.45 ± 13.21, which was significantly lower than the HA group (73.61 ± 7.85) and the M2c‐Exos@HA group (85.49 ± 9.93) at 4 weeks. There was no significant difference between the HA group and the M2c‐Exos@HA group in the MRI index at 4 weeks. However, the MRI index of the M2c‐Exos@HA group (81.37 ± 6.91) was significantly higher than the degeneration group (43.97 ± 4.74) and HA group (58.29 ± 6.62) at 8 weeks.

According to H&E staining and safranin O‐fast green staining, we measured the histological degeneration score of the IVDD to evaluate the degree of IVDD between the different groups 4 and 8 weeks after injection (Figure 5c,d). Four weeks after injection, the degeneration group exhibited a disordered ECM structure with a large number of infiltrating inflammatory cells around the NPCs. The HA and M2c‐Exos@HA groups showed moderate infiltration of inflammatory cells and less ECM loss than the degeneration group. However, these two groups had similar degenerated morphology, ECM preservation, and histological degeneration scores (HA group: 7.24 ± 0.92, M2c‐Exos@HA group: 7.18 ± 1.62) at 4 weeks, although they were better than the degeneration group. Eight weeks after injection, the structural disorder and loss of ECM in the degeneration group deteriorated and were replaced by a large amount of fibrous tissue. Although the HA group and the M2c‐Exos@HA group also showed some fibrosis, the structure and ECM of the NP were well preserved. The M2c‐Exos@HA group exhibited a more orderly and clearer structure of the NP, less fibrosis, and more retention of the ECM than the HA group. Although the histological scores of the HA group and the M2c‐Exos@HA group were significantly lower than the degeneration group (12.77 ± 1.02), the M2c‐Exos@HA group (7.26 ± 0.86) was significantly lower than the HA group (8.61 ± 1.37). These results indicated that M2c‐Exos@HA did not have a significant therapeutic effect in the early stage of degeneration, but it significantly delayed the progression of IVDD in long‐term degeneration.

**FIGURE 6:**
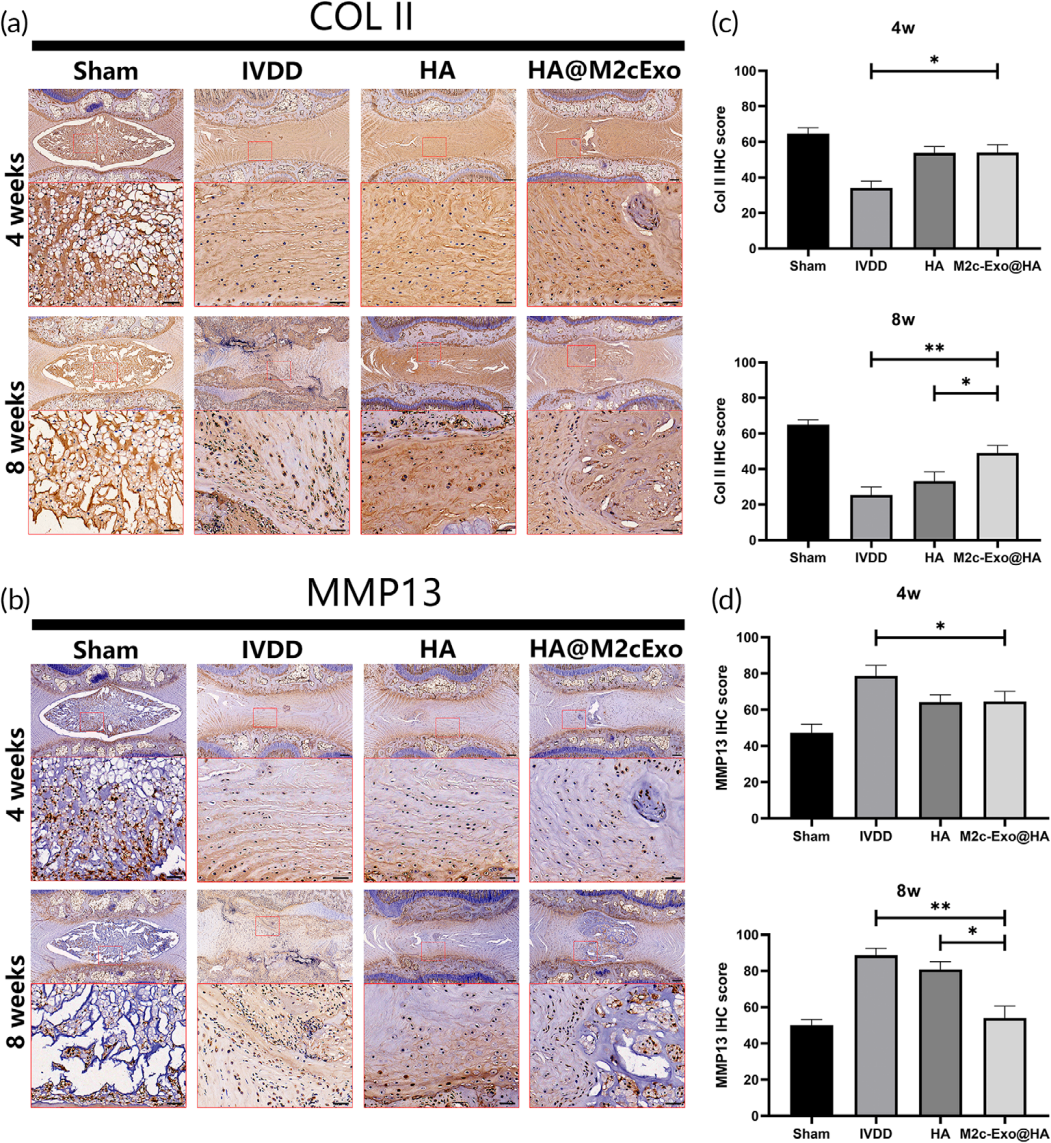
Administration of the M2c‐Exos@HA hydrogel improved the synthesis of Col II and inhibited the secretion of MMP13 in IVDD in vivo. (a, b) IHC staining of Col II and MMP13 in different groups 4 and 8 weeks after implantation. (c, d) Quantification of Col II and MMP13 IHC staining in different groups. *P < 0.05, **P < 0.01. Scale bar: 200 μm.

We performed immunohistochemical staining to evaluate the expression of Col IIand MMP13 in degenerated intervertebral discs after intervention. As shown in Figure 6a–d, the IHC score of collagen type II in the HA and M2c‐Exos@HA groups was significantly higher than the degeneration group 4 weeks after intervention, and the IHC score of MMP13 was significantly lower than the degeneration group. However, there was no significant difference in the IHC scores of Col II and MMP13 between the M2c‐Exos@HA and HA groups. Notably, the IHC scores of Col II in the degeneration and HA groups were significantly lower than the M2c‐Exos@HA group 8 weeks after intervention, and the scores of MMP‐13 in these two groups were significantly higher than the M2c‐Exos@HA group. These results suggest that the M2c‐Exos@HA hydrogel improved the secretion of ECM proteins, inhibited metalloproteinases, and promoted the regeneration of degenerated NP in the long‐term process.

### 3.7 4D‐LFP proteomic analysis of M2c‐Exos‐treated NPCs

**FIGURE 7:**
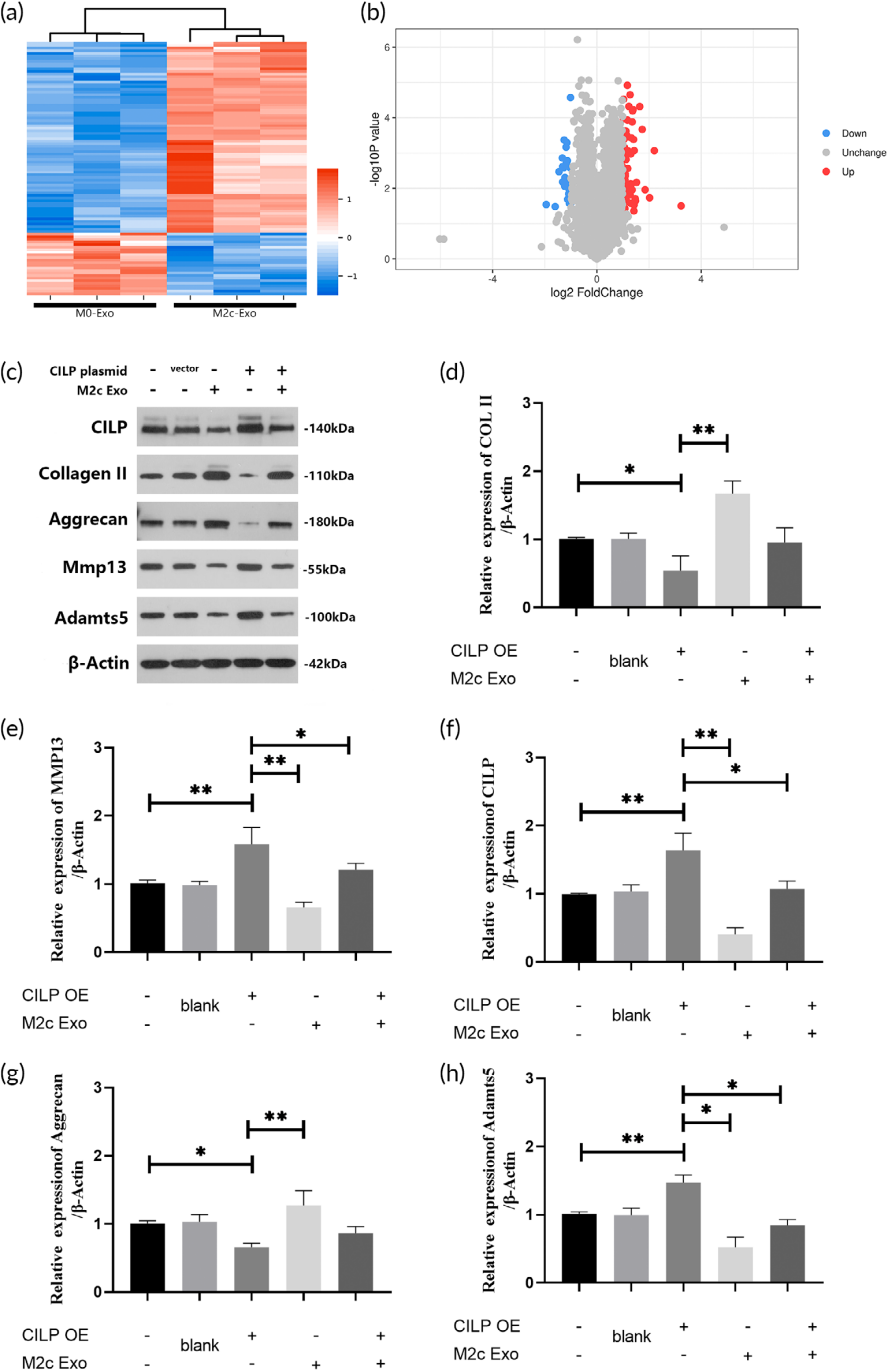
Proteomic analysis revealed that M2c‐Exoss promoted extracellular matrix (ECM) synthesis in nucleus pulposus cells (NPCs) by regulating cartilage intermediate layer protein (CILP). (a) Heatmap of the 4D‐LFP proteome in NPCs treated with M2c‐Exoss. (b) Volcano plot showing NPCs treated with M2c‐Exoss. (c–e) Western blot analysis detecting the expression of CILP, Col II, aggrecan, MMP13, and ADAMTS5 in NPCs treated with CILP‐overexpressing plasmid or M2c‐Exoss. *p < 0.05, **p < 0.01.

To explore the mechanism by which M2c‐Exoss promote regeneration of IVDD via regulation of the ECM anabolism and catabolism of NPCs, we used 4D‐LFP label‐free proteomics analysis to detect the high‐throughput changes of protein in NPCs after intervention with M2c‐Exoss. As shown by heatmaps (Figure 7a), the expression patterns of proteins in groups treated with different exosomes (M0‐Exos and M2c‐Exoss) were distinctly clustered, which suggested significant M2c‐Exos‐induced dysregulation of protein expression in NPCs. We set |fold‐change| >2 and a BH‐adjusted *p* value <0.05 as the criteria to screen for differentially expressed proteins in M2c‐Exos‐treated NPCs. As shown by the volcano plot (Figure 7b), 98 proteins in NPCs were significantly changed. Twenty‐four proteins were down‐regulated, and 74 proteins were up‐regulated. GO and KEGG analyses were performed on differentially expressed proteins to further classify their functions and identify the signaling pathways involved, which may reflect the influence of M2c‐Exoss on the metabolic phenotype and signal transduction of NPCs to a certain extent. The differentially expressed proteins were primarily enriched in integrin binding, extracellular space, oxidoreductase activity, the cytokine–cytokine interacting pathway, and the NF‐κB pathway (Figure S4), which suggests that M2c‐Exoss affect the above‐mentioned biological functions and pathways of NPCs.

### 3.8 M2c‐Exoss promoted the synthesis of the NPC matrix by regulating CILP

The present study further screened the down‐regulated proteins in NPCs treated with M2c‐Exoss and found that cartilage intermediate layer protein (CILP) was the most significantly inhibited (|fold‐change| = 2.74, adj‐*p* value = 0.003). Several studies found that CILP overexpression was associated with IVDD.^42^, ^43^, ^44^ Therefore, we used a plasmid‐based approach to overexpress CILP in NPCs that were simultaneously treated with M2c‐Exoss to verify whether CILP played a critical role in M2c‐Exos‐mediated metabolic regulation of the ECM of NP. Western blot results showed that treatment with M2c‐Exoss dramatically inhibited the expression of CILP in NPCs (Figure 7c–h). Overexpression of CILP dominantly down‐regulated Col II and aggrecan and up‐regulated MMP13 and ADAMTS5. Notably, the combined intervention of M2c‐Exoss partially offset the above‐mentioned effects of CILP overexpression on ECM proteins and metalloproteinases. Based on these findings, we selected CILP as the key molecule mediating M2c‐Exos‐induced anabolic and catabolic ECM rebalance in the NP and focused on CILP to further examine the upstream regulatory molecules in M2c‐Exoss and the downstream ECM metabolic pathways in NPCs.

### 3.9 CILP was targeted and inhibited by M2c‐Exos‐transferred miR‐124

**FIGURE 8:**
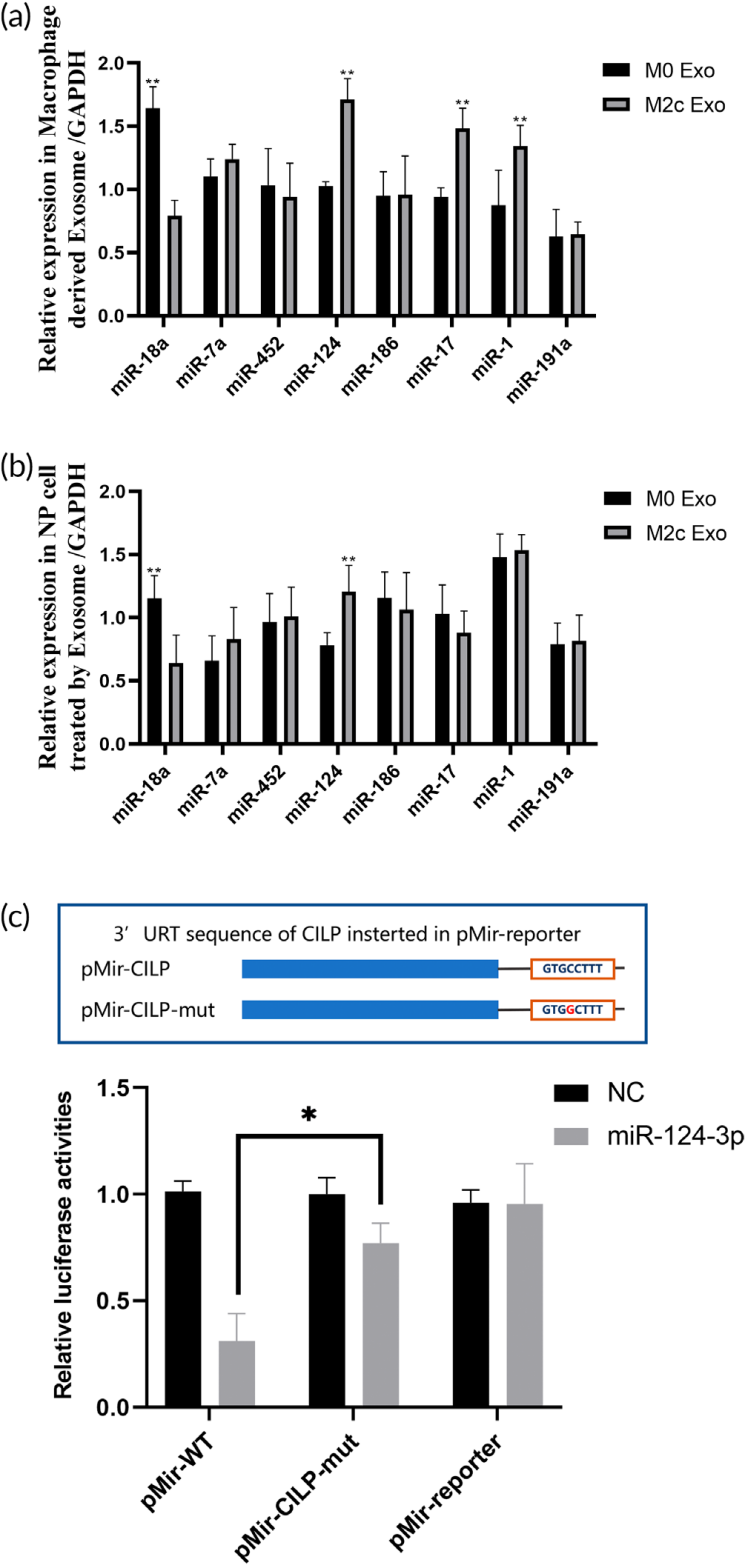
M2c‐Exoss inhibited cartilage intermediate layer protein (CILP) expression in nucleus pulposus cells (NPCs) by transferring miR‐124. (a) Comparison of miRNAs potentially targeting CILP in M2c‐Exoss and M0‐Exos using qPCR. (b) Comparison of miRNAs potentially targeting CILP in NPCs treated with M2c‐Exoss and M0‐Exos using qPCR. (c) Luciferase reporter assay demonstrating that miR‐124 specifically bound to the 3′ UTR of CILP mRNA. *p < 0.05, **p < 0.01.

Exosomes generally regulate the phenotype of receptor cells by delivering active substances, such as miRNA. Due to the heterogeneity of exosomal RNAs, the different miRNAs may have opposite effects on the phenotypic regulation of recipient cells.^26^, ^45^ Therefore, accurately predicting and verifying the function of exosomal RNA is critical in research on exosome‐induced regeneration. Based on the findings mentioned above, we used CILP as the core of the M2c‐Exos‐dependent mechanism and further used the TargetScan database to predict the potential miRNAs targeting CILP mRNA. Eight miRNAs, miR‐18a, miR‐7a, miR‐452, miR‐124, miR‐186, miR‐17, miR‐1, and miR‐191a were screened depending on the context score. Validation using qPCR showed that mir‐124 was significantly enriched in M2c‐Exoss compared to M0‐Exos (Figure 8a). The expression of mir‐124 in M2c‐Exos‐treated NPCs was significantly higher than M0‐Exo‐treated NPCs (Figure 8b). These results indicated that M2c‐Exoss evidently delivered miR‐124 into NPCs.

MiRNAs bind to specific sequences at the 3′ end of targeted mRNAs and promote the degradation of target mRNAs, which inhibits the expression of target proteins.^46^ To further investigate whether miR‐124 targeted the 3′ end of CILP mRNA and inhibited its expression, we constructed a dual‐luciferase reporter system (including normal and variant sequences of CILP) and transfected it into HEK293 cells. As shown in Figure 8c, the luciferase activity was significantly reduced after intervention with miR‐124. When the CILP sequence was mutated, the effect of miR‐124 on luciferase activity was partially inhibited. This finding indicates that miR‐124 targets and degrades CILP mRNA.

**FIGURE 9:**
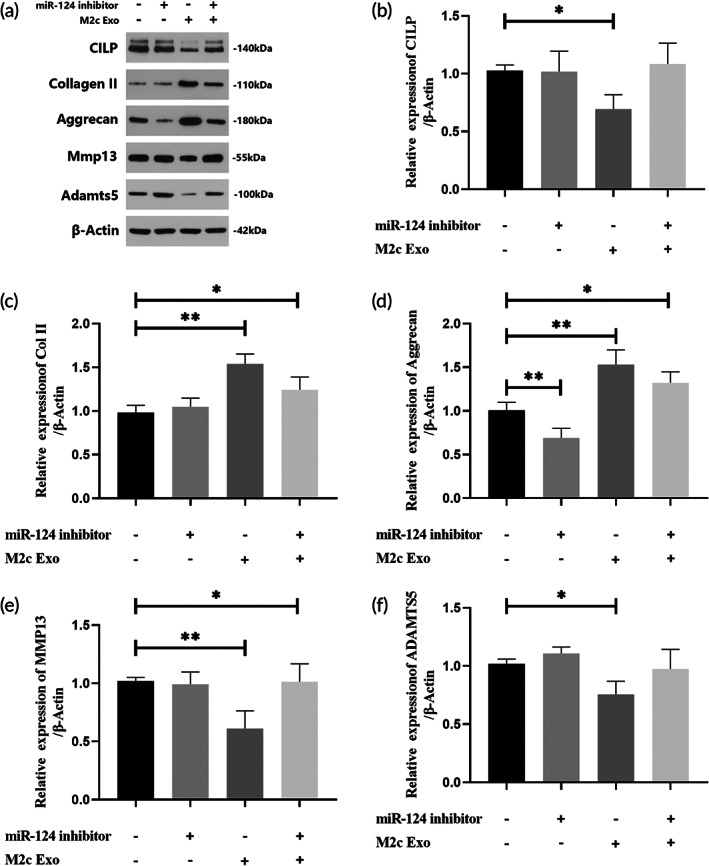
MiR‐124 transferred by M2c‐Exoss promoted extracellular matrix (ECM) synthesis in nucleus pulposus cells (NPCs) by inhibiting cartilage intermediate layer protein (CILP). (a) Western blot analysis detecting the expression of CILP, Col II, aggrecan, MMP13, and ADAMTS5 in NPCs treated with miR‐124 inhibitors or M2c‐Exoss. (b–f) Quantification of the Western blot analysis mentioned above. *p < 0.05, **p < 0.01.

To further demonstrate that miR‐124 mediated the regulation of M2c‐Exos on CILP in NPCs, we detected the expression of CILP, ECM protein, and metalloproteinase in NPCs under the combined intervention of an miR‐124 inhibitor and M2c‐Exos intervention. Western blotting showed (Figure 9a) that the miR‐124 inhibitor did not significantly affect the expression of CILP in NP cells. However, when combined with M2c‐Exoss, the miR‐124 inhibitor partially offset the inhibitory effect of M2c‐Exoss on CILP expression and reversed the pro‐expressing effect of M2c‐Exoss on Col II and aggrecan and its down‐regulating effect on MMP‐13 and ADAMTS5 (Figure 9b–f). These results suggested that M2c‐Exoss inhibited CILP in NPCs by delivering miR‐124 to improve the anabolism and catabolism of ECM in the NP.

### 3.10 M2c‐Exoss activated the TGF‐β/Smad 3 pathway indirectly by suppressing CILP

**FIGURE 10:**
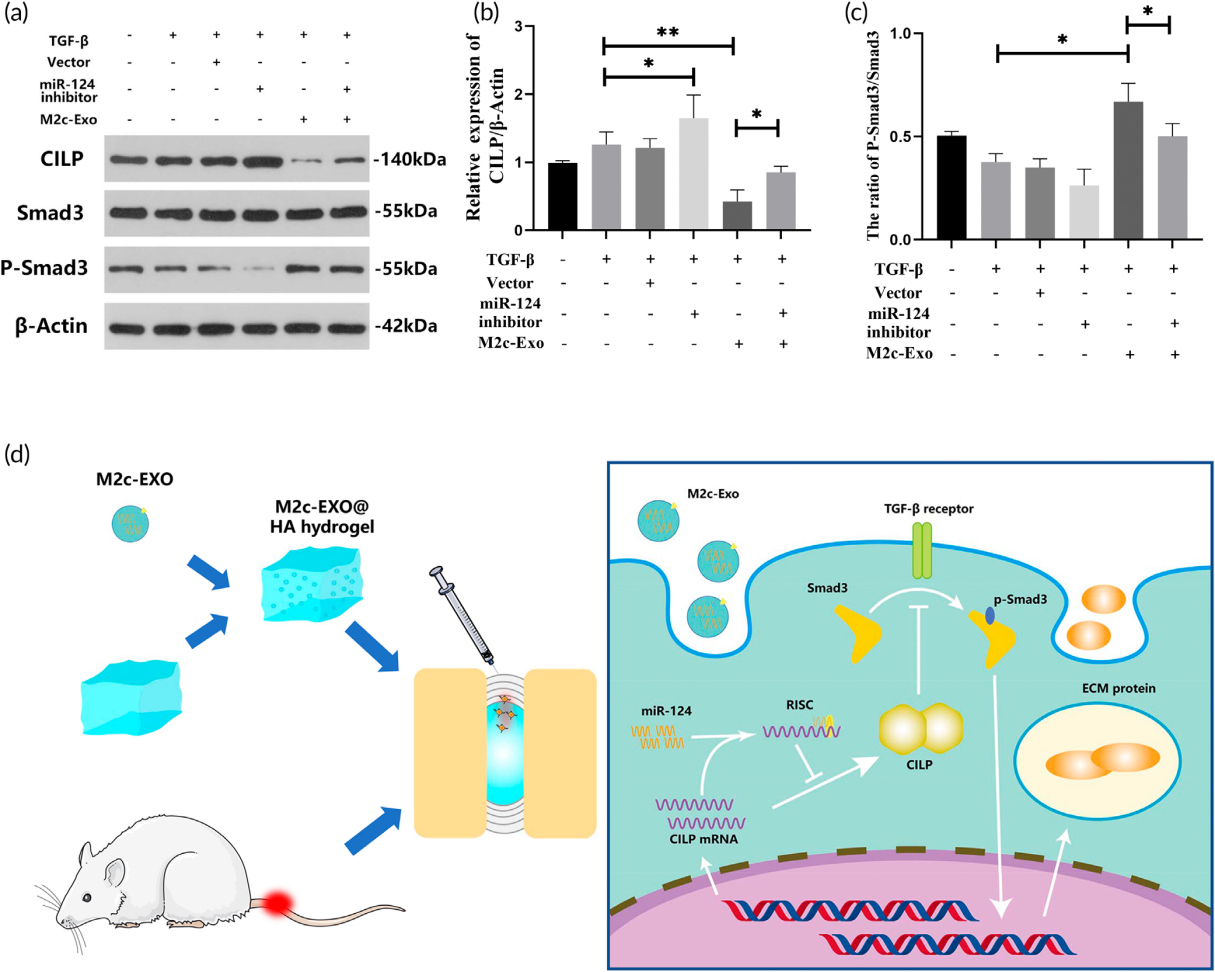
Inhibition of cartilage intermediate layer protein (CILP) by M2c‐Exoss activated extracellular matrix (ECM) synthesis in nucleus pulposus cells (NPCs) by enhancing the TGF‐β/Smad3 pathway. (a) Western blot analysis detecting the expression of CILP, Smad3, and P‐Smad3 in NPCs treated with TGF‐β, miR‐124 inhibitors or M2c‐Exoss. (b, c) Quantification of the Western blot analysis mentioned above. (d) Schematic illustration of the mechanism by which M2c‐Exoss promote the synthesis of ECM in NPCs via the miR‐124/CILP/TGF‐β axis. *p < 0.05, **p < 0.01.

It has been reported that the pro‐degenerating effects of CILP on the NP we related to the hindrance of the TGF‐β signaling pathway.^43^ Therefore, we used the miR‐124 inhibitor, M2c‐Exos (150 μg/mL) and TGF‐β (10 ng/mL) to jointly intervene in NPCs and detected the activity of the TGF‐β signaling pathway using Western blotting. As shown in Figure 10a–c, M2c‐Exoss significantly up‐regulated the phosphorylation of Smad3, but the miR‐124 inhibitor had no significant influence on the phosphorylation of Smad3. Intervention with the miR‐124 inhibitor significantly impaired the phosphorylation effect of M2c‐Exoss on Smad3. These results indicated that M2c‐Exoss indirectly promoted the phosphorylation of Smad3 and enhanced the transduction of the TGF‐β pathway by inhibiting the expression of CILP protein.

## 4 DISCUSSION

The imbalance of ECM metabolism and inflammatory regulation in intervertebral discs is the fundamental mechanism of IVDD. Treatments to regulate this disorder and restore the normal metabolic microenvironment of the NP are crucial to the repair of IVDD. Therefore, an immune‐modulating‐based intervention strategy for IVDD was introduced in the present study (Figure 10d). The exosome‐mediated intercellular transfer of miR‐124 between M2c macrophages and NPCs regulated the CILP/TGF‐β axis and subsequently alleviated IVDD by enhancing synthesis and inhibiting degeneration of the ECM.

Autoimmune responses to the NP play a critical role in promoting IVDD, in which a variety of pro‐inflammatory cytokines and cells inhibit the anabolism of NP matrix proteins (e.g., proteoglycan and type II collagen) and induce secretion of matrix metalloproteinases (e.g., MMP and ADAMTS families), which worsen the inflammatory response and matrix degeneration.^47^, ^48^, ^49^ M2‐polarizing macrophages promote tissue regeneration by antagonizing the pro‐inflammatory factors mentioned above. Therefore, different subtypes of M2 macrophages (M2a, M2b, and M2c) probably have reversal effects on NP degeneration. M2a macrophages, a subtype of M2 polarization induced by IL‐4, aggravated the degeneration of the NP matrix by secreting the anti‐inflammatory cytokine IL‐13.^28^ This contradictory effect of M2a macrophages in IVDD may be attributed to their inaction on tissue remodeling.^21^ Moreover, M2b macrophages are characterized as simultaneously having inflammatory regulating function and effects of promoting tumor growth and invasion.^50^ After being induced by combined immune complexes as well as TLR or IL‐1R agonists, M2b macrophages produce IL1, IL6, IL10, and TNFα, which are harmful factors for intervertebral discs.^51^ Therefore, we selected M2c macrophages, which are induced by IL‐10 and TGF‐β and function as a remodeling phenotype, as a potential pivot to investigate their intercellular communicating effects on NPCs. M2c macrophages promote the vitality of NP cells and ameliorate ECM dysmetabolism. Therefore, the relevant mechanisms by which M2c macrophages stabilize the metabolism of the NP ECM should be further explored in future research.

Exosomes are an important carrier for the intercellular exchange of information and effective substitutes for simulating the bio‐regulating functions of original cells.^52^ The modulating potential of exosomes from various cells for the regenerative microenvironment has been widely explored in the field of intervertebral disc rebuilding. Exosomes from bone marrow mesenchymal stem cells inhibited the assembly of the NLRP3‐mediated inflammasome and alleviated apoptosis of NP cells induced by oxidative stress and inflammation.^53^, ^54^ Urine‐derived stem cells promote the proliferation of NP cells and the secretion of matrix protein via the exosomal transport of protein MATN3 to NP cells.^55^ Exosomes from notochords cells prestimulated by pressure inhibited vascularization and inflammation via the miR‐140/Wnt/β‐catenin regulatory axis.^56^ M2c macrophages possess functions similar to stem cells in immune modulation and homeostasis of the microenvironment.^57^ It is rationally presumed that exosomes mediate the intercellular regulation of M2c macrophages on NPCs to enhance vitality and matrix protein synthesis. Therefore, the present study further demonstrated this presumption in vitro and in vivo. Exosomes are intraluminal vesicles that are precursors of exosomes, which are generally derived from intracellular in‐folding of the membrane mediated by ceramide.^58^ The small molecule compound GW4869 selectively antagonizes ceramides and blocks the formation of intraluminal vesicles, which terminates the secretion of exosomes.^59^ Therefore, GW4869 was selected to block the exosomal secretion of M2c macrophages in the present study and indirectly demonstrate the presumption mentioned above. As shown in the results, M2c macrophages pretreated with GW4869 promoted the proliferation and migration of NPCs. However, pretreatment with GW4869 significantly antagonized the upregulation of matrix proteins (e.g., Col II and aggrecan) and the inhibition of matrix proteases (e.g., MMP‐13 and ADAMTS5) induced by M2c macrophages. These results suggest that M2c macrophages stimulate the anabolism of the ECM and inhibit its degeneration by transferring exosomes to NPCs. According to this clue, we treated NP cells with different concentrations of M2c macrophage exosomes and found that M2c macrophage exosomes significantly up‐regulated the expression of Col II and aggrecan in the NP cells and down‐regulated the expression of MMP‐13 and ADAMTS5, with an optimal effective concentration of 150 μg/mL. These results indicate that M2c macrophage‐derived exosomes effectively ameliorate the degeneration of the NP matrix by regulating its metabolism instead of enhancing the vitality of NPCs in vitro.

Compared to the uncomplicated environment in vitro, the in vivo microenvironment combined with multiple factors restricts external exosomes from exerting pro‐regenerative efficacy.^60^ Because of the rapid clearance and nonspecific internalization of exosomes by the microcirculation and other cells, the effective duration of exosomes is reduced significantly in vivo and barely covers the entire process of regeneration.^61^ The main strategy to deliver exosomes accurately and adequately in vivo is the use of biomaterials as effective carriers of exosomes, which ensures the stability and long‐term release of exosomes in specific damaged tissue.^62^ Hydrogels are the most commonly used bioactive material for the loading and delivery of exosomes to promote tissue regeneration.^63^, ^64^ The present study selected HA hydrogel as the carrier of M2c macrophage exosomes because the internal structure of HA after gelatinization was similar to the NP tissue, which is propitious for M2c macrophage exosomes to exert modulating effects. The decomposition of HA hydrogel lasts more than 1 month, which makes the release of M2c macrophage exosomes sufficiently cover the inflammatory process of the intervertebral disc.^65^, ^66^ In the construction of the HA hydrogel, we used the gelation strategy of directly mixing HA powder and a suspension of M2c macrophage exosomes without a crosslinker to avoid unknown effects caused by the crosslinker. As shown in the present study, HA hydrogel effectively loaded M2c macrophage exosomes and prolonged the continuous release of M2c macrophage exosomes in coccygeal intervertebral discs of rats for up to 28 days. Therefore, HA hydrogels are capable of providing favorable conditions for M2c macrophage exosomes in vivo. According to the results of MRI, histological staining, and IHC in the present study, HA hydrogel loaded with M2c macrophage exosomes showed an excellent capability of preserving the height, hydration, and histological structure of the coccygeal intervertebral disc after puncture‐induced degeneration in advanced stages after intervention (8 weeks). As shown by the IHC results, the M2c‐Exos@HA hydrogel also significantly promoted the expression of collagen II and inhibited the expression of MMP13, which improved the anabolism and remodeling of the NP ECM. However, the M2c‐Exos@HA hydrogel was not superior to the HA hydrogels alone in terms of reversing the IVDD mentioned above at the early stage after implantation (4 weeks). Given the effects of attenuating inflammation at early stage of IVDD,^67^, ^68^ HA hydrogels probably covered the antidegeneration effects of M2c‐Exos. While HA hydrogels degraded completely at 4 weeks after intervention, its blanketing effects on M2c‐Exos vanished, which further resulting in the reveal of M2c‐Exos therapeutic effects. Therefore, M2c macrophage exosomes combined with HA hydrogels have long‐term therapeutic effects of improving the metabolism of the NP matrix and promoting IVDD regeneration in vivo.

4D‐LFQ proteomics was used to analyze the differential proteins in NP cells treated with M2c‐Exoss, and we found that CILP, which was significantly down‐regulated by M2c macrophage exosome intervention, was a potential key molecule mediating the metabolic regulatory effects of M2c‐Exoss on the ECM of NPCs. CILP is a chondrocyte‐secreted protein that is primarily deposited in the interterritorial matrix of articular cartilage.^69^, ^70^ The abnormal expression of CILP is highly associated with the progression of IVDD.^42^, ^71^ Transgenic mice overexpressing CILP exhibit a tendency toward IVDD.^43^ Long‐term cyclic tension induces the expression of CILP protein in human NPCs, which resulted in the downregulation of Col II and aggrecan.^72^ The present study found that M2c macrophage exosomes were potentially dependent on CILP downregulation to produce long‐term improvement of matrix anabolism, synthesis of Col II and aggrecan, and inhibition of matrix metalloproteinases in NPCs.

A variety of noncoding RNAs contained in exosomes may mediate the regulation of M2c macrophages in the metabolism of NPCs via targeted regulation of CILP. To accurately determine the up‐stream regulatory molecules of CILP in M2c‐Exoss, we used TargetScan data to perform backward prediction of miRNAs and subsequently identified miR‐124, which potentially acts on CILP. MiR‐124 was also identified. According to qPCR validation and luciferase reporter assays, miR‐124 was delivered by M2c‐Exoss into NPCs and bound to a sequence of the 3′ end of CILP mRNA, which inhibited its translation. This finding indicates that miR‐124 transferred by M2c‐Exoss specifically inhibits the expression of CILP depending on the classical miRNA‐induced mechanism of mRNA silencing.^46^ In subsequent rescue experiment of present study, the reversal effects of miR‐124 inhibitor on ECM metabolic regulation of M2c‐Exoss further demonstrated that miR‐124 promoted the synthesis of ECM proteins and inhibited metalloproteinases by regulating the expression of CILP in NP cells. However, it was intriguing that the single miR‐124 inhibitor had no influence on CILP expression. We speculate that there might be a reason accounts for this paradoxical phenomenon. Accordingly, CILP is regulated by various miRNAs and endogenous signals, such as miR‐330‐5p and miR‐542‐3p, which are endogenously expressed in NPCs and synergistically maintaining the low expression level of CILP in NPCs.^73^, ^74^ Only inhibition of miR‐124 can hardly increase CLIP level.

The molecular mechanism by which CILP promotes disc degeneration is significantly related to its inhibitory function of the TGF‐β/Smad3 pathway, which is critical in maintaining the natural metabolism of the NP matrix.^43^, ^44^ The CILP protein has similar domains as the TGF‐β receptor, and CILP hinders the activation of the TGF‐β receptor by competitively binding to TGF‐β and inhibiting the phosphorylation of Smad3.^75^ Activation of the TGF‐β/Smad3 pathway also promotes the expression of CILP protein.^76^ This phenomenon is the negative feedback regulatory mechanism of the TGF‐β/Smad3 signaling pathway mediated by CILP. The present study confirmed that Smad3 phosphorylation was enhanced in NPCs under intervention with M2c‐Exoss, which suggests that M2c macrophage exosomes regulate the activation of the TGF‐β/Smad3 pathway. After blocking the mRNA silencing effects of mir‐124, the up‐regulated CILP significantly inhibited TGF‐β‐induced phosphorylation of Smad3, which led to inhibition of ECM protein expression and enhancement of metalloproteinase secretion in NPCs. Additional intervention with M2c‐Exoss in NPCs reversed the blockade of Smad3 phosphorylation and the imbalance in ECM metabolism caused by the miR‐124 inhibitor. Therefore, the above‐mentioned phenomenon accounts for the molecular mechanism of M2c‐Exoss in metabolic improvement of the NP matrix, in which miR‐124 was delivered to indirectly activate the TGF‐β/SMad3 pathway via the targeted regulation of CILP. Notably, the proliferation of NPCs is activated by the TGF‐β/Smad3 pathway.^77^ However, M2c macrophage exosomes had no significant influence on the proliferation of NP cells with activation of the TGF‐β/Smad3 pathway in the present study. This phenomenon was likely attributed to the undiscovered proliferative regulation of NP cells by other underlying bioactive molecules from M2c‐Exoss, which should be explored in future research.

There are several limitations in the present study. First, an acupuncture model of a rat coccyx disc cannot thoroughly mimic the pathological process of IVDD. IVDD in humans is associated with adaptive changes in the NP and cartilage endplate induced by long‐term multiaxial pressure. The acupuncture model of rat coccyx discs cannot reflect the degeneration induced by mechanical loading. Breakage of the annulus fibrosus using acupuncture leads to man‐made changes in the mechanical microenvironment of the intervertebral disc rather than natural degenerating processes.^78^ Therefore, to strictly simulate the mechanical mechanism of IVDD, it is urgent to explore new animal models of IVDD in future research. Second, proteomic analysis identified CILP as the core of the mechanism by which M2c‐Exoss regulated the metabolism of the NP matrix. MiR‐124 from M2c‐Exoss was backward speculated and verified as the upstream regulator of CILP. This research strategy was conducive to quickly identifying the CILP‐centered regulating axis comprised exosomal miR‐124 and the TGF‐β/Smad3 pathway, by which M2c‐Exoss affected the phenotype of NP cells. However, the forward strategy based on M2c‐Exos transcriptomic analysis was used to screen the regulatory noncoding RNAs in M2c‐Exoss more extensively than the above‐mentioned backward research. Therefore, other potential noncoding RNAs regulating NP metabolism should be mined in future research.

In conclusion, exosomes from M2c macrophages may serve as therapeutic agents for IVDD via the miR‐124/CILP/TGF‐β/Smad3 regulatory axis. HA hydrogel may be used as an effective carrier for the long‐term conservation and sustained release of M2c macrophage exosomes in degenerated discs in vivo. The present study demonstrated that exosomes derived from M2c macrophages combined with biomaterials were a prospective strategy for regulating intervertebral disc matrix metabolism and promoting degenerative regeneration.

## AUTHOR CONTRIBUTIONS

**Yi Liu:** Conceptualization (lead); data curation (lead); formal analysis (lead); software (lead); validation (equal); visualization (equal); writing – original draft (lead); writing – review and editing (lead). **Mintao Xue:** Conceptualization (equal); data curation (equal); formal analysis (equal); investigation (equal); methodology (equal); software (equal); writing – original draft (equal); writing – review and editing (equal). **Yaguang Han:** Conceptualization (equal); data curation (equal); formal analysis (equal); investigation (equal); methodology (equal); software (equal); writing – original draft (equal); writing – review and editing (equal). **Yucai Li:** Data curation (supporting); investigation (supporting); methodology (supporting); software (supporting); writing – original draft (supporting). **Bing Xiao:** Data curation (supporting); investigation (supporting); methodology (supporting); validation (supporting); visualization (supporting). **Weiheng Wang:** Data curation (supporting); investigation (supporting); methodology (supporting); software (supporting); visualization (supporting). **Jiangming Yu:** Conceptualization (equal); funding acquisition (equal); project administration (equal); resources (equal); supervision (equal). **Xiaojian Ye:** Funding acquisition (lead); project administration (lead); resources (lead); supervision (lead).

## FUNDING INFORMATION

This study was supported by National Key R&D Program of China (Grant No. 2020YFC2008404), National Natural Science Foundation of China (Grant No. 82102605), Natural Science Foundation of Shanghai, China (Grant No. 80ZR1469800), Interdisciplinary Program of Shanghai Jiao Tong University (Grant No. YG2021ZD34).

## CONFLICT OF INTEREST STATEMENT

The authors declare no conflicts of interest.

## Supporting information


**Data S1:** Supporting Information

## Data Availability

The datasets used and/or analyzed during the current study are available from the corresponding authors on reasonable request.
