# Supplementary material for: Exosomes from M2c macrophages alleviate intervertebral disc degeneration by promoting synthesis of the extracellular matrix via MiR‐124/CILP/TGF‐β
Source: Bioeng Transl Med. 2023 Feb 16;8(6):e10500. doi: 10.1002/btm2.10500 (PMC10658595; doi:10.1002/btm2.10500)
Supplement: Supplementary file 1 — Data S1: Supporting Information [file BTM2-8-e10500-s001.pdf]

Supplementary materials

Exosomes from M2c Macrophages Alleviate Intervertebral Disc Degeneration by Promoting Synthesis of the Extracellular Matrix via MiR-124/CILP/TGF-β

Yi Liu[1,2#], Mintao Xue[3#], Yaguang Han[3#], Yucai Li[2], Weiheng Wang[3], Bing Xiao[3], Jiangming Yu[2*], Xiaojian Ye[2*]

1 Department of Orthopedic, Tianjin First Central Hospital, Tianjin, P.R. China
2 Department of Orthopedics, Tongren Hospital, Shanghai Jiao Tong University School of Medicine, Shanghai, P.R. China.
3 Department of Orthopaedics, Second Affiliated Hospital of Naval Medical University, Shanghai, P. R. China

#Yi Liu, Mintao Xue, and Yaguang Han contributed equally to this work

*Corresponding author:
Jiangming Yu, Department of Orthopedics, Tongren Hospital, Shanghai Jiao Tong University School of Medicine, No. 1111, Xianxia Road, Shanghai 200336, P.R. China, Tel: +86 21 52039999, Fax: +86 21 52039963; E-mail: yjm_st@163.com.
Xiaojian Ye, Department of Orthopedics, Tongren Hospital, Shanghai Jiao Tong University School of Medicine, No. 1111, Xianxia Road, Shanghai 200336, P.R. China, Tel: +86 21 52039999, Fax: +86 21 52039963; E-mail: yexj2002@163.com.

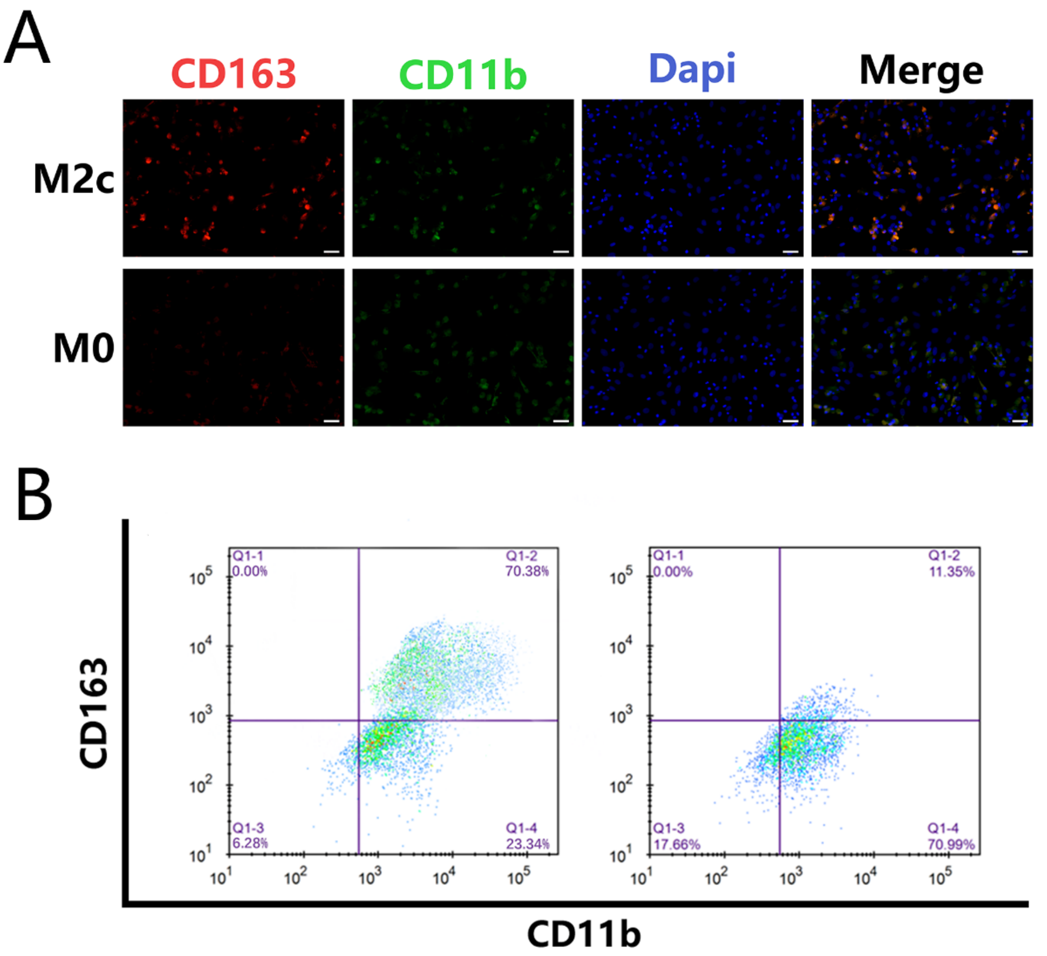

Fig. S1 Identification of M2c polarization of macrophage induced with IL-10. (A) immunofluorescent staining of CD11b (general marker of macrophage) and CD163 (specific marker of M2c macrophage). (B) Flow cytometry analysis on CD11b and CD163 of M2c macrophage. Scale bar = 50 μm.

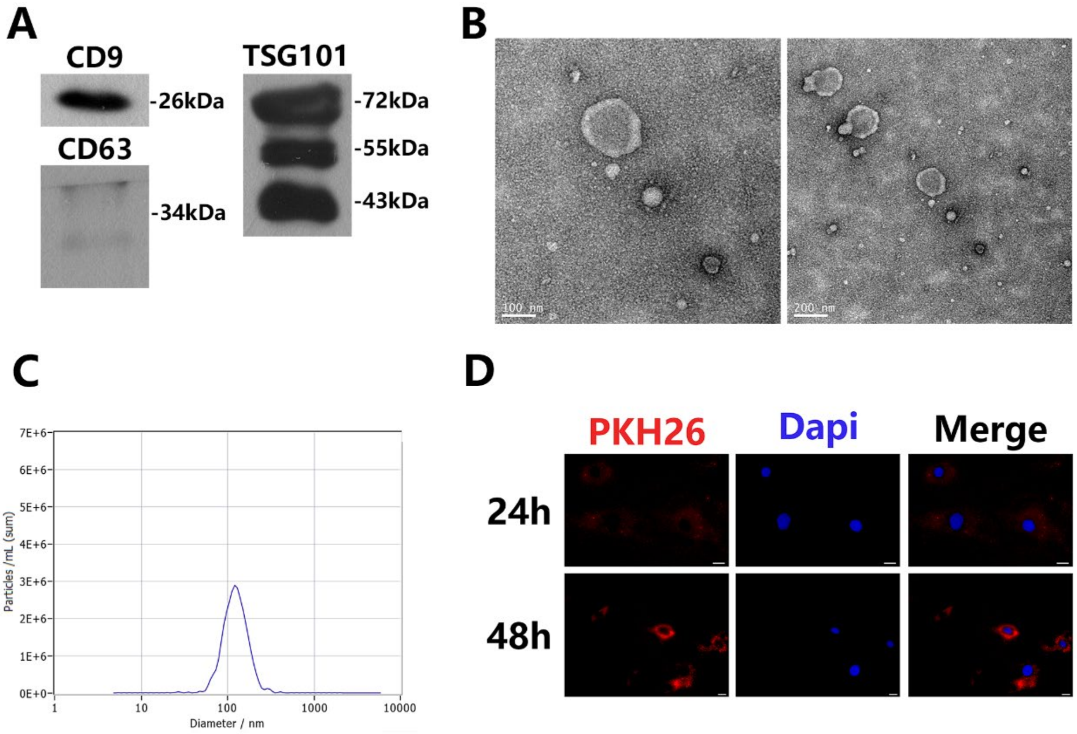

Fig. S2 Identification of exosomes from M2c macrophage induced with IL-10. (A) Western blot of exosomal makers including CD9, CD63, TSG101. (B) Representative images of M2c-Exo under TEM. Scale bar: 100 and 200 nm. (C) The size distribution of M2c-Exo detected by NTA. Internalization of PKH26 labeled M2c-Exo by NPCs. Scale bars = 20 μm.

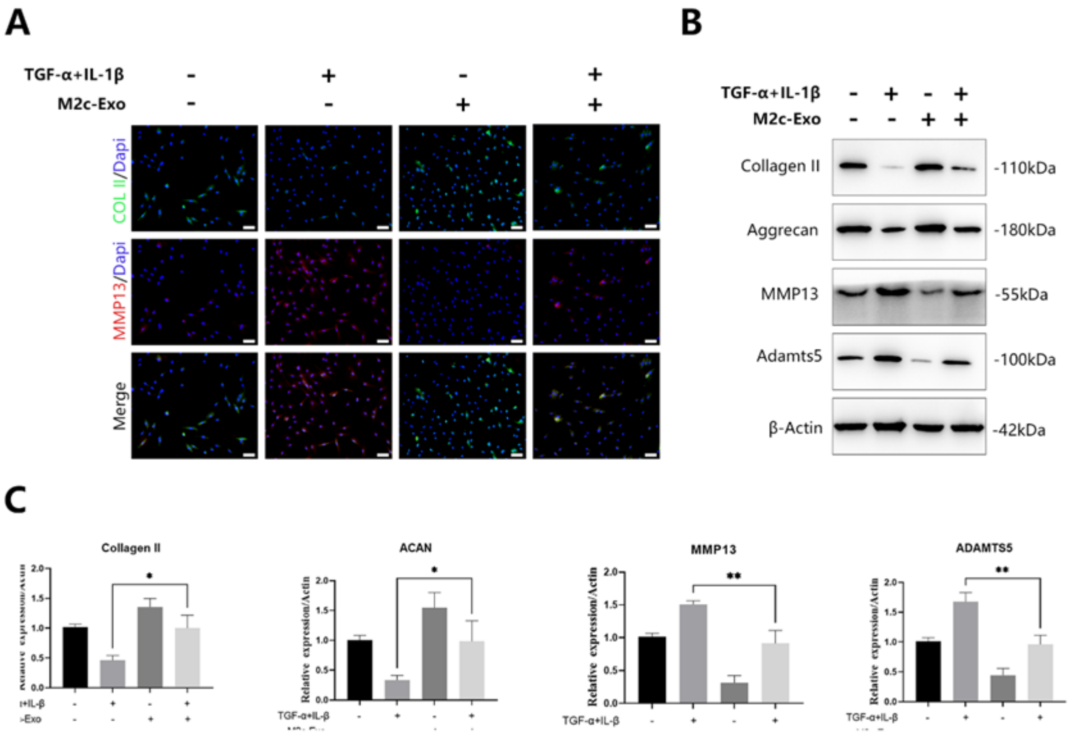

Fig. S3 M2c-Exos improved ECM metabolism of NPCs in treatment of TNF-α and IL-1β. (A, B) The alternation of Col II, aggrecan, MMP13 and ADAMTS5 in NPCs incubated in conditions of TNF-α and IL-1β with or without M2c-Exos (150 μg/ml) shown by immunofluorescent staining and Western blotting. Scale bar = 100 μm. (C) Quantification for immunofluorescent staining and Western blotting. $*p < 0.05$, $**p < 0.01$.

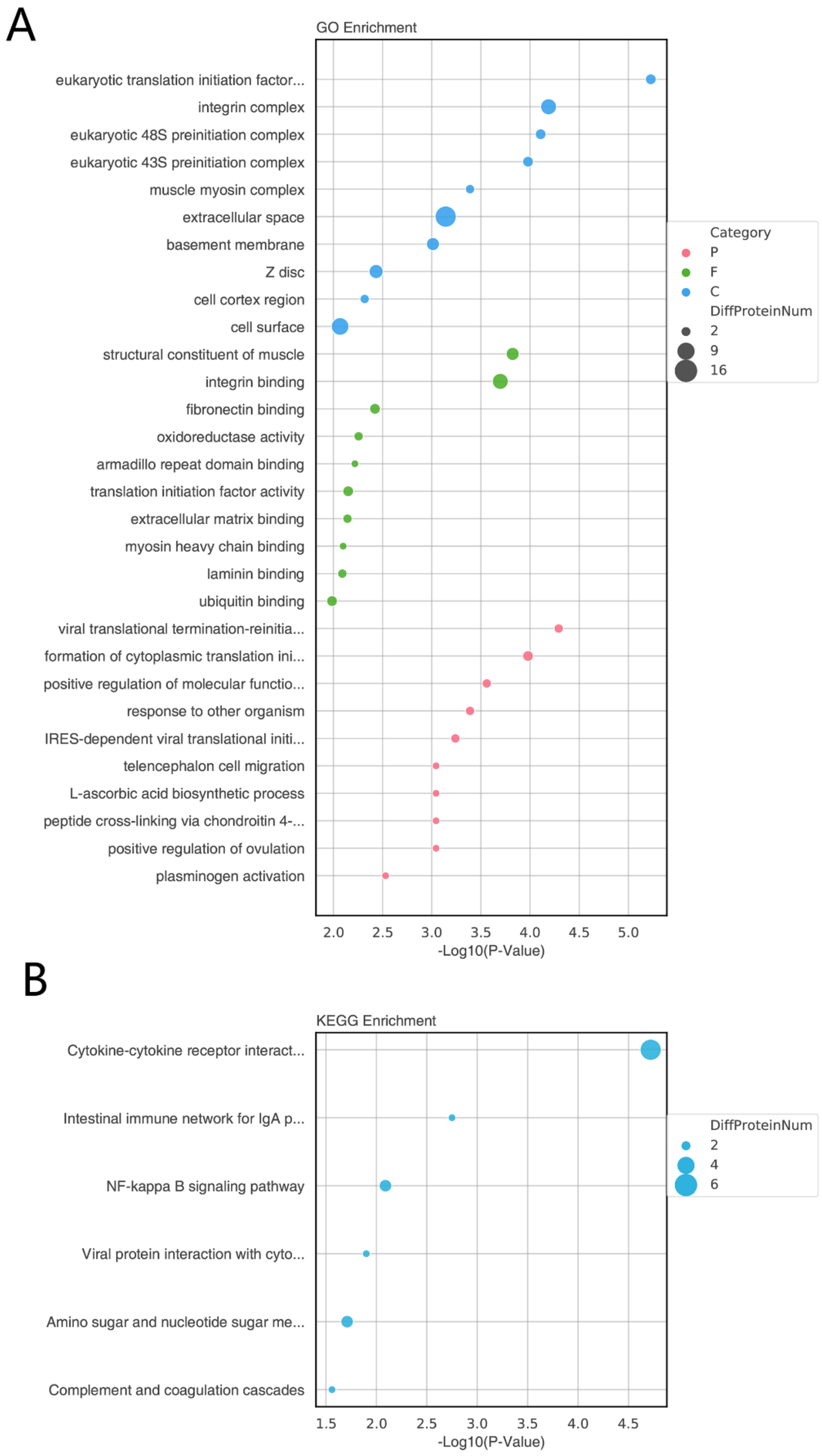

Fig. S4 Gene Ontology analysis (A) and KEGG analysis (B) of different expressed proteins in NPCs treated with M2c-Exos or M0-Exo.

Table S1. Primers fo miRNAs

miR-124	Forward	5’-aggcacgcggtga-3’
	Reverse	5’-tccagttttttttttttttttggca-3’
miR-18a	Forward	5’-ttgttctaaggtgcatctagtgcagatagtgaagtag actagcatctactgccctaagtgctccttct-3’
	Reverse	5’-ggtccagttttttttttttttttgac-3’
miR-7a	Forward	5’-gccctgtctggaagactagtgattttgttgttgtgtctgtgtc-3’
	Reverse	5’-gtccagttttttttttttttttgatgtg-3’
miR-452	Forward	5’-caggctaaacacttacaactgt-3’
	Reverse	5’-ggtccagttttttttttttttttgca-3’
miR-186	Forward	5’-tttccaaagaattctccttttgggcttt ctcattttattttaagcccaaaggtgaatttttgggaagt-3’
	Reverse	5’-gtccagttttttttttttttttagctc-3’
miR-17	Forward	5’-caaagtgcttacagtgcaggtagcttcttgaga-3’
	Reverse	5’-tccagttttttttttttttttcatggt-3’
miR-1	Forward	5’-gagcacatacttctttatgtaccca tatgaacatagaatgctatggaatg-3’
	Reverse	5’-ggtccagttttttttttttttttacct-3’
miR-191a	Forward	5’-cgggcaacggaatcccaaaa gcagctgttgtctccagagcattccag-3’
	Reverse	5’-gtccagttttttttttttttttaggca-3’
